# Physics-informed learning of governing equations from scarce data

**DOI:** 10.1038/s41467-021-26434-1

**Published:** 2021-10-21

**Authors:** Zhao Chen, Yang Liu, Hao Sun

**Affiliations:** 1 Department of Civil and Environmental Engineering, Northeastern University, Boston, MA 02115 USA; 2 Department of Mechanical and Industrial Engineering, Northeastern University, Boston, MA 02115 USA; 3 Gaoling School of Artificial Intelligence, Renmin University of China, 100872 Beijing, China; 4 Beijing Key Laboratory of Big Data Management and Analysis Methods, 100872 Beijing, China; 5 Department of Civil and Environmental Engineering, MIT, Cambridge, MA 02139 USA

**Keywords:** Computational science, Scientific data

## Abstract

Harnessing data to discover the underlying governing laws or equations that describe the behavior of complex physical systems can significantly advance our modeling, simulation and understanding of such systems in various science and engineering disciplines. This work introduces a novel approach called physics-informed neural network with sparse regression to discover governing partial differential equations from scarce and noisy data for nonlinear spatiotemporal systems. In particular, this discovery approach seamlessly integrates the strengths of deep neural networks for rich representation learning, physics embedding, automatic differentiation and sparse regression to approximate the solution of system variables, compute essential derivatives, as well as identify the key derivative terms and parameters that form the structure and explicit expression of the equations. The efficacy and robustness of this method are demonstrated, both numerically and experimentally, on discovering a variety of partial differential equation systems with different levels of data scarcity and noise accounting for different initial/boundary conditions. The resulting computational framework shows the potential for closed-form model discovery in practical applications where large and accurate datasets are intractable to capture.

## Abstract (web summary)

Recovery of underlying governing laws or equations describing the evolution of complex systems from data can be challenging if dataset is damaged or incomplete. The authors propose a learning approach which allows to discover governing partial differential equations from scarce and noisy data.

## Introduction

Current practices on modeling of complex dynamical systems have been mostly rooted in the use of ordinary and/or partial differential equations (ODEs, PDEs) that govern the system behaviors. These governing equations are conventionally obtained from rigorous first principles such as the conservation laws or knowledge-based phenomenological derivations. However, there remain many real-world complex systems underexplored, whose analytical descriptions are undiscovered and parsimonious closed forms of governing equations are unclear or partially unknown. Luckily, observational datasets become increasingly rich and offer an alternative of distilling the underlying equations from data. Harnessing data to uncover the governing laws or equations can significantly advance and transform our modeling, simulation, and understanding of complex physical systems in various science and engineering disciplines. For example, obtaining mathematical equations that govern the evolution of sea ice from observational data (e.g., satellite remote sensing images) brings distinct benefits for better understanding and predicting the growth, melt and movement of the Arctic ice pack. Distilling an explicit formulation from field sensing data (e.g., Doppler radar recordings) will accelerate more accurate prediction of weather and climate patterns. Recently, advances in machine learning theories, computational capacity, and data availability kindle significant enthusiasm and efforts towards data-driven discovery of physical laws and governing equations^1–13^.

Pioneering contributions by Bongard and Lipson^1^ and Schmidt and Lipson^2^ leveraged stratified symbolic regression and genetic programming to successfully distil the underlying differential equations that govern nonlinear system dynamics from data. However, this elegant approach does not scale up well with the dimensionality of the system, is computationally expensive, and might suffer from overfitting issues. Recently, an impressive breakthrough made by Brunton et al.^5^ leads to an innovative sparsity-promoting approach called sparse identification of nonlinear dynamics (SINDy), which selects dominant candidate functions from a high-dimensional nonlinear function space based on sparse regression to uncover parsimonious governing equations, ODEs in particular. The sparsity was achieved by a sequential threshold ridge regression (STRidge) algorithm which recursively determines the sparse solution subjected to hard thresholds^5,6^. Such an approach is capable of balancing the complexity and accuracy of identified models and thus results in parsimony. SINDy has drawn tremendous attention in the past few years, leading to variant algorithms with applications to identify projected low-dimensional surrogate models in the form of first-order ODEs, alternatively with linear embedding^8,10^, for a wide range of nonlinear dynamical systems, such as fluid flows^14,15^, structural systems^16,17^, biological and chemical systems^18–20^, active matter^21^, predictive control of nonlinear dynamics^22^, multi-time-scale systems^23^, a predator–prey system^24^, and stochastic processes^25^, just naming a few among many others. There are also a number of other extensions of SINDy that discover implicit dynamics^18,26^, incorporate physics constraints^14^, and embed random sampling to improve the robustness to noise for sparse discovery of high-dimensional dynamics^27^. The convergence and error estimate analyses^28^ theoretically sustain the family of SINDy approaches.

The sparsity-promoting paradigm has been later extended for the data-driven discovery of spatiotemporal systems governed by PDEs, e.g., the PDE-FIND algorithm^6,7^, where the library of candidate functions is augmented by incorporating spatial partial derivative terms. This method has been further investigated or improved to, for example, obtain parametric PDEs from data^29^, discover PDEs enhanced by Bayesian inference^30^ and gene expression programming^31^, identify diffusion and Navier-Stokes equations based on molecular simulation^32^, and learn PDEs for biological transport models^33^. Nevertheless, a critical bottleneck of the SINDy framework, especially for the data-driven discovery of PDEs, lies in its strong dependence on both quality and quantity of the measurement data, since numerical differentiation is required to compute the derivatives in order to construct governing equation(s). Especially, the use of finite difference or filtering to calculate derivatives leads to a pivotal challenge that reduces the algorithm robustness. This specially limits the applicability of SINDy in its present form to scenarios given highly incomplete, scarce and noisy data. It is notable that variational system identification^9^ shows satisfactory robustness of calculating derivatives based on isogeometric analysis for discovering the weak form of PDEs. However, such an approach doesn’t scale down well with respect to the fidelity of available data. Another work^34^ shows that weak formulation can significantly improve the discovery robustness against noise, but requires careful design of test functions, which is intractable for high-dimensional spatiotemporal systems.

Automatic differentiation^35^ is well-posed to address the above issue, which has been proven successful in physics-informed neural networks (PINN) for forward and inverse analyses of nonlinear PDEs^36–40^. In particular, the deep neural network (DNN) is used to approximate the solution constrained by both the PDE(s) and a small amount of available data. PINN has attracted increasing attention for tackling in a wide range of scientific problems such as fluid flows^39,40^, vortex-induced vibrations^41^, cardiovascular systems^42^, among many others, when the explicit form of PDEs is known. Recently, the important work by Raissi^43^ introduced a deep hidden physics model for data-driven modeling of spatiotemporal dynamics based on sparse data, where the unknown underlying physics characterized by possible PDE terms is weakly imposed and implicitly learned by an auxiliary neural network. Nevertheless, the resulting model is still a “black box” and lacks sufficient interpretability since the closed-form governing equations cannot be uncovered. Latest studies^44,45^ show the potential of using DNNs and automatic differentiation to obtain closed-form PDEs, from noisy data, in a constrained search space with a pre-defined library of PDE terms; yet, false-positive identification occurs due to the use of less rigorous sparse regression along with DNN training. In fact, simultaneously optimizing the DNN parameters and sparse PDE coefficients, while accurately enforcing sparsity, is non-trivial and remains a significant challenge in closed-form PDE discovery.

To this end, we leverage these advances and leap beyond to present a novel PINN-SR method (i.e., PINN with sparse regression), possessing salient features of interpretability and generalizability, to discover governing PDEs of nonlinear spatiotemporal systems from scarce and noisy data. Our approach integrates the strengths of DNNs for rich representation learning, automatic differentiation for accurate derivative calculation as well as *ℓ*_0_ sparse regression to tackle the fundamental limitation of existing methods that scale poorly with data noise and scarcity. In particular, the paper involves two methodological contributions: (1) a “root-branch” network, constrained by unified underlying physics, that is capable of dealing with a small number of multi-datasets coming from different initial/boundary conditions, and (2) a simple, yet effective, alternating direction training strategy for optimization of heterogeneous parameters, i.e., DNN trainable parameters and sparse PDE coefficients. The efficacy and robustness of our method are demonstrated on a variety of PDE systems, based on both numerical and experimental datasets.

## Results

### PINN with sparse regression for PDE discovery

We consider a multi-dimensional spatiotemporal system whose governing equations can be described by a set of nonlinear, coupled, parameterized PDEs in the general form given by

1 $${{{{{{{{\bf{u}}}}}}}}}_{t}+{{{{{{{\mathcal{F}}}}}}}}\left[{{{{{{{\bf{u}}}}}}}},{{{{{{{{\bf{u}}}}}}}}}^{2},\ldots ,{\nabla }_{{{{{{{{\bf{x}}}}}}}}}{{{{{{{\bf{u}}}}}}}},{\nabla }_{{{{{{{{\bf{x}}}}}}}}}^{2}{{{{{{{\bf{u}}}}}}}},{\nabla }_{{{{{{{{\bf{x}}}}}}}}}{{{{{{{\bf{u}}}}}}}}\cdot {{{{{{{\bf{u}}}}}}}},\ldots ;{{{{{{{\boldsymbol{\lambda }}}}}}}}\right]={{{{{{{\bf{p}}}}}}}}$$

where ${{{{{{{\bf{u}}}}}}}}={{{{{{{\bf{u}}}}}}}}({{{{{{{\bf{x}}}}}}}},t)\in {{\mathbb{R}}}^{1\times n}$ is the multi-dimensional latent solution (dimension = *n*) while **u**_*t*_ is the first-order time derivative term; *t* ∈ [0, *T*] denotes time and **x** ∈ Ω specifies the space; ${{{{{{{\mathcal{F}}}}}}}}[\cdot ]$ is a complex nonlinear functional of **u** and its spatial derivatives, parameterized by ***λ***; ∇ is the gradient operator with respect to **x**; **p** = **p**(**x**, *t*) is the source term (note that, in many common cases, **p = 0** represents no source input to the system). The PDEs are also subjected to initial and boundary conditions (I/BCs), if known, denoted by ${{{{{{{\mathcal{I}}}}}}}}[{{{{{{{\bf{x}}}}}}}}\in {{\Omega }},t=0;{{{{{{{\bf{u}}}}}}}},{{{{{{{{\bf{u}}}}}}}}}_{t}]=0$ and ${{{{{{{\mathcal{B}}}}}}}}[{{{{{{{\bf{x}}}}}}}}\in \partial {{\Omega }};{{{{{{{\bf{u}}}}}}}},{\nabla }_{{{{{{{{\bf{x}}}}}}}}}{{{{{{{\bf{u}}}}}}}}]=0$. For systems that obey Newton’s second law of motion (e.g., **u**_*tt*_ in wave equations), the governing PDEs can be written in a state-space form of Eq. (1) by defining **v** = {**uu**_*t*_} as the solution variable. Our objective is to find the closed form of ${{{{{{{\mathcal{F}}}}}}}}[\cdot ]$ from available spatiotemporal measurements which are assumed to be incomplete, scarce and noisy commonly seen in real-world applications (e.g., when data capture is very costly or the data itself is sparse in nature). We assume that the physical law is governed by only a few important terms which can be selected from a large-space library of candidate functions, where sparse regression can be applied^5–7^. Inherent in this assumption leads to a reformulation of Eq. (1) in the following (assuming zero or unknown source for simplicity):

2 $${{{{{{{{\bf{u}}}}}}}}}_{t}={{{{{{{\boldsymbol{\phi }}}}}}}}{{{{{{{\mathbf{\Lambda }}}}}}}}$$

Here, ${{{{{{{\boldsymbol{\phi }}}}}}}}={{{{{{{\boldsymbol{\phi }}}}}}}}({{{{{{{\bf{u}}}}}}}})\in {{\mathbb{R}}}^{1\times s}$ is an extensive library of symbolic functions consisting of many candidate terms, e.g., constant, polynomial, and trigonometric terms with respect to each spatial dimension^6,7^, assembled in a row vector given by ${{{{{{{\boldsymbol{\phi }}}}}}}}=\{1,{{{{{{{\bf{u}}}}}}}},{{{{{{{{\bf{u}}}}}}}}}^{2},\ldots ,{{{{{{{{\bf{u}}}}}}}}}_{x},{{{{{{{{\bf{u}}}}}}}}}_{y},\ldots ,{{{{{{{{\bf{u}}}}}}}}}^{3}\odot {{{{{{{{\bf{u}}}}}}}}}_{xy},\ldots ,\sin ({{{{{{{\bf{u}}}}}}}}),\ldots \}$, where ⊙ represents the element-wise Hadamard product; *s* denotes the total number of candidate terms in the library; the subscripts in the context of {*x*, *y*, *z*} depict the derivatives; ${{{{{{{\mathbf{\Lambda }}}}}}}}\in {{\mathbb{R}}}^{s\times n}$ is the sparse coefficient matrix (only the active candidate terms in ***ϕ*** have non-zero values), e.g., ${{{{{{{\mathbf{\Lambda }}}}}}}}=\left[{{{{{{{{\boldsymbol{\lambda }}}}}}}}}^{u}\,{{{{{{{{\boldsymbol{\lambda }}}}}}}}}^{v}\,{{{{{{{{\boldsymbol{\lambda }}}}}}}}}^{w}\right]\in {{\mathbb{R}}}^{s\times 3}$ for **u** = {*u*, *v*, *w*}. If there is an unknown source input, the candidate functions for **p** can also be incorporated into ***ϕ*** for discovery (see Supplementary Note 3.1). Thus, the discovery problem can then be stated as: given the spatiotemporal measurement data ${{{{{{{{\mathcal{D}}}}}}}}}_{u}$, find sparse **Λ** such that Eq. (2) holds.

**Fig. 1:**
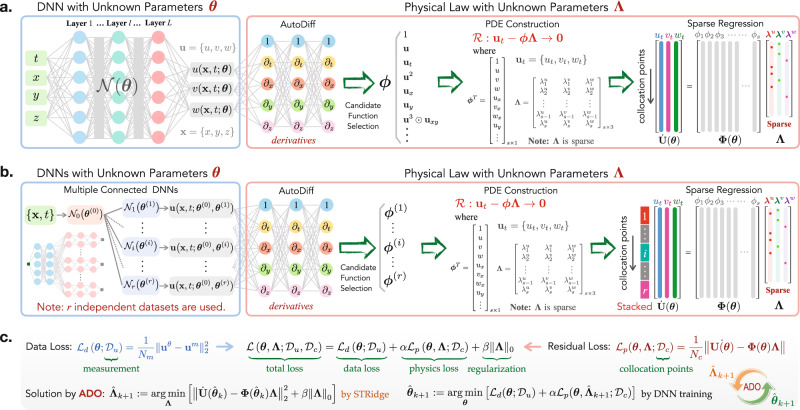
Schematic architecture of the framework of PINN-SR for data-driven discovery of PDE(s). **a** the network for one dataset from a single I/BC, **b** the “root-branch” network for *r* ≥2  independent datasets from multiple I/BCs, and **c** schematic for training the networks based on alternating direction optimization. The network consists of two components: a DNN governed by the trainable parameters ***θ***, which maps the spatiotemporal coordinates {**x**, *t*} to the latent solution **u** = {*u*, *v*, *w*}, and the physical law described by a set of nonlinear PDEs, which are formed by the derivative candidate functions ***ϕ*** parameterized by the unknown sparse coefficients **Λ**. Note that, for the case of multiple independent datasets, the libraries ***ϕ***^(*i*)^ are concatenated to build ***ϕ*** for constructing the unified governing PDE(s). The total loss function ${{{{{{{\mathcal{L}}}}}}}}({{{{{{{\boldsymbol{\theta }}}}}}}},{{{{{{{\mathbf{\Lambda }}}}}}}};{{{{{{{{\mathcal{D}}}}}}}}}_{u},{{{{{{{{\mathcal{D}}}}}}}}}_{c})$ is composed of the data loss ${{{{{{{{\mathcal{L}}}}}}}}}_{d}({{{{{{{\boldsymbol{\theta }}}}}}}},{{{{{{{{\mathcal{D}}}}}}}}}_{u})$, the physics loss $\alpha {{{{{{{{\mathcal{L}}}}}}}}}_{p}({{{{{{{\boldsymbol{\theta }}}}}}}},{{{{{{{\mathbf{\Lambda }}}}}}}};{{{{{{{{\mathcal{D}}}}}}}}}_{c})$, and the *ℓ*_0_ regularization term *β*∥**Λ**∥_0_ that promotes the sparsity. Here, *α* and *β* denote the relative weighting of the loss functions, while ${{{{{{{{\mathcal{D}}}}}}}}}_{u}$ and ${{{{{{{{\mathcal{D}}}}}}}}}_{c}$ represent the measurement data and collocation samples respectively. Note that the physics loss, in a residual form, is only evaluated on the spatiotemporal collocation samples. The colored dots in the sparse coefficients matrix (or vector) on the right denote non-zero values. Simultaneous optimization of the unknown parameters {***θ***, **Λ**} leads to both the trained DNN for inference of the data-driven full-field solution and the discovered parsimonious closed-form PDEs.

We present a new PINN-SR paradigm to simultaneously model the system response and identify the parsimonious closed form of the governing PDE(s). The innovative algorithm architecture of this method is shown in Fig. 1, where datasets sampled from two different I/BC scenarios are considered: (1) one dataset from a single I/BC and (2) *r* ≥ 2 independent datasets from multiple I/BCs. For the case of single dataset, we interpret the latent solution **u** by a DNN (denoted by ${{{{{{{\mathcal{N}}}}}}}}$), namely, **u**^*θ*^ = **u**(**x**, *t*; ***θ***), where ***θ*** represents the DNN trainable parameters including weights and biases, as shown in Fig. 1a. When multiple independent datasets are available, a “root-branch” DNN depicted in Fig. 1b is designed to approximate the latent solutions **u**_*i*_ (*i* = 1, …, *r*) corresponding to different I/BCs, viz., ${{{{{{{{\bf{u}}}}}}}}}_{i}^{\theta }={{{{{{{\bf{u}}}}}}}}\left({{{{{{{\bf{x}}}}}}}},t;{{{{{{{{\boldsymbol{\theta }}}}}}}}}^{(0)},{{{{{{{{\boldsymbol{\theta }}}}}}}}}^{(i)}\right)$, where ***θ***^(0)^ and ***θ***^(*i*)^ denote the trainable parameters of the root layers ${{{{{{{{\mathcal{N}}}}}}}}}^{(0)}$ and the branch layers ${{{{{{{{\mathcal{N}}}}}}}}}^{(i)}$, respectively. Noteworthy, the I/BCs are unnecessarily either known a priori or measured since the measurement data already reflects the specific I/BC (e.g., there exists a one-to-one mapping between the I/BC and the PDE solution). The DNN essentially plays a role as a nonlinear functional to approximate the latent solution with the data loss function ${{{{{{{{\mathcal{L}}}}}}}}}_{d}({{{{{{{\boldsymbol{\theta }}}}}}}};{{{{{{{{\mathcal{D}}}}}}}}}_{u})$. With automatic differentiation where derivatives on **u** are evaluated at machine precision, the library of candidate functions ***ϕ***^*θ*^ can be computed from the DNN. For the case of multiple independent datasets, the libraries ***ϕ***^(*i*)^ resulted from the branch nets are concatenated to build ***ϕ***^*θ*^ for constructing the unified governing PDE(s). Thus, the sparse representation of the reconstructed PDE(s) can be written in a residual form, namely, ${{{{{{{{\bf{{{{{{{{\mathcal{R}}}}}}}}}}}}}}}}}^{\theta }:= {{{{{{{{\bf{u}}}}}}}}}_{t}^{\theta }-{{{{{{{{\boldsymbol{\phi }}}}}}}}}^{\theta }{{{{{{{\mathbf{\Lambda }}}}}}}}\to {{{{{{{\bf{0}}}}}}}}$, where ${{{{{{{{\bf{{{{{{{{\mathcal{R}}}}}}}}}}}}}}}}}^{\theta }\in {{\mathbb{R}}}^{1\times n}$ denotes the PDE residuals. The basic concept is to adapt both the DNN trainable parameters ***θ*** and the PDE coefficients **Λ** such that the neural network can fit the measurement data while satisfying the constraints defined by the underlying PDE(s). The PDE residuals will be evaluated on a large number of collocation points ${{{{{{{{\mathcal{D}}}}}}}}}_{c}={\{{{{{{{{{\bf{x}}}}}}}}}_{i},{t}_{i}\}}_{i = 1}^{{N}_{c}}$, randomly sampled in the spatiotemporal space, leading to the residual physics loss function ${{{{{{{{\mathcal{L}}}}}}}}}_{p}({{{{{{{\boldsymbol{\theta }}}}}}}},{{{{{{{\mathbf{\Lambda }}}}}}}};{{{{{{{{\mathcal{D}}}}}}}}}_{c})$. When multiple I/BCs are considered, the measurement data and the collocation points will be stacked when calculating the data loss and the physics loss (based on a unified physics residual formulation ${{{{{{{{\bf{{{{{{{{\mathcal{R}}}}}}}}}}}}}}}}}^{\theta }\to {{{{{{{\bf{0}}}}}}}}$).

The total loss function for training the overall PINN-SR network is thus composed of the data loss ${{{{{{{{\mathcal{L}}}}}}}}}_{d}$, the residual physics loss ${{{{{{{{\mathcal{L}}}}}}}}}_{p}$ and a regularization term, expressed as

3 $${{{{{{{\mathcal{L}}}}}}}}({{{{{{{\boldsymbol{\theta }}}}}}}},{{{{{{{\mathbf{\Lambda }}}}}}}};{{{{{{{{\mathcal{D}}}}}}}}}_{u},{{{{{{{{\mathcal{D}}}}}}}}}_{c})={{{{{{{{\mathcal{L}}}}}}}}}_{d}({{{{{{{\boldsymbol{\theta }}}}}}}};{{{{{{{{\mathcal{D}}}}}}}}}_{u})+\alpha {{{{{{{{\mathcal{L}}}}}}}}}_{p}({{{{{{{\boldsymbol{\theta }}}}}}}},{{{{{{{\mathbf{\Lambda }}}}}}}};{{{{{{{{\mathcal{D}}}}}}}}}_{c})+\beta \parallel {{{{{{{\mathbf{\Lambda }}}}}}}}{\parallel }_{0}$$

where *α* is the relative weighting of the residual physics loss function; *β* is the regularization parameter; ∥ ⋅ ∥_0_ represents the *ℓ*_0_ norm. Optimizing the total loss function can produce a DNN that can not only predict the data-driven full-field system response, but also uncover the parsimonious closed-form PDE(s), i.e., $\{{{{{{{{{\boldsymbol{\theta }}}}}}}}}^{\star },{{{{{{{{\mathbf{\Lambda }}}}}}}}}^{\star }\}:={\arg \min }_{\{{{{{{{{\boldsymbol{\theta }}}}}}}},{{{{{{{\mathbf{\Lambda }}}}}}}}\}}\left[{{{{{{{\mathcal{L}}}}}}}}({{{{{{{\boldsymbol{\theta }}}}}}}},{{{{{{{\mathbf{\Lambda }}}}}}}};{{{{{{{{\mathcal{D}}}}}}}}}_{u},{{{{{{{{\mathcal{D}}}}}}}}}_{c})\right]$, where {***θ***^⋆^, **Λ**^⋆^} denote the optimal set of parameters. Noteworthy, the total loss function has an implicit complex form, and thus, directly solving the optimization problem is highly intractable since the *ℓ*_0_ regularization makes this problem *np*-hard. To address this challenge, we present an alternating direction optimization (ADO) algorithm that divides the overall optimization problem into a set of tractable subproblems to sequentially optimize the trainable parameters, as shown in Fig. 1c. Pre-training of PINN-SR is conducted before running the ADO algorithm for discovery, by simply replacing ∥**Λ**∥_0_ in Eq. (3) with ∥**Λ**∥_1_ where brute-force gradient-based optimization for both ***θ*** and **Λ** becomes applicable. The *ℓ*_1_-regularized pre-training can accelerate the convergence of ADO by providing an admissible “initial guess”. More detailed formulation and algorithm description are found in Methods and Supplementary Note 1.

The synergy of DNN and sparse regression results in the following outcome: the DNN provides accurate modeling of the latent solution, its derivatives and possible candidate function terms as a basis for constructing the governing PDE(s), while the sparsely represented PDE(s) in turn constraints the DNN modeling and projects correct candidate functions, eventually turning the measured system into closed-form PDE(s).

### Discovery of benchmark PDEs with single dataset

**Fig. 2:**
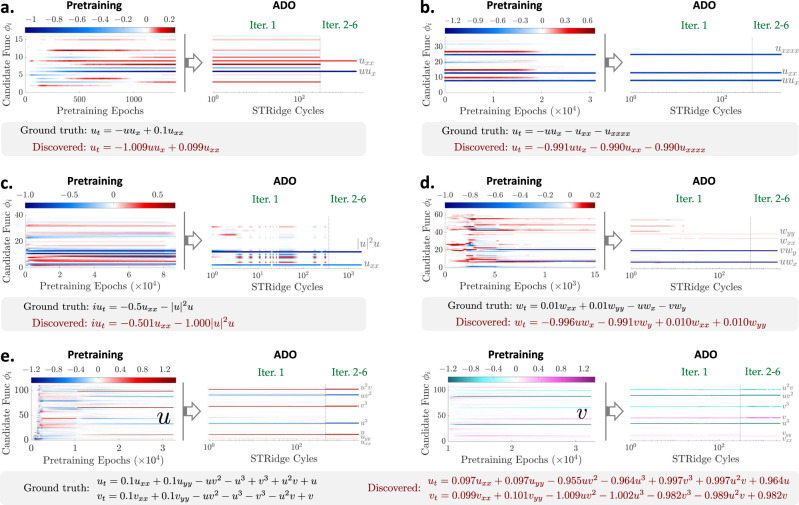
Discovery of selected benchmark PDEs for sparsely sampled measurement data with 10% noise. **a** Discovered Burgers' equation: evolution of the sparse coefficients ${{{{{{{\mathbf{\Lambda }}}}}}}}\in {{\mathbb{R}}}^{16\times 1}$ for 16 candidate functions ${{{{{{{\boldsymbol{\phi }}}}}}}}\in {{\mathbb{R}}}^{1\times 16}$ used to form the PDE, where the color represents the coefficient value. **b** Discovered KS equation: Evolution of the sparse coefficients ${{{{{{{\mathbf{\Lambda }}}}}}}}\in {{\mathbb{R}}}^{36\times 1}$ for 36 candidate functions ${{{{{{{\boldsymbol{\phi }}}}}}}}\in {{\mathbb{R}}}^{1\times 36}$. **c** Discovered nonlinear Schrödinger equation: evolution of the sparse coefficients ${{{{{{{\mathbf{\Lambda }}}}}}}}\in {{\mathbb{R}}}^{40\times 1}$ for the candidate functions ${{{{{{{\boldsymbol{\phi }}}}}}}}\in {{\mathbb{R}}}^{1\times 40}$. **d** Discovered NS equation: evolution of the sparse coefficients ${{{{{{{\mathbf{\Lambda }}}}}}}}\in {{\mathbb{R}}}^{60\times 1}$ for 60 candidate functions ${{{{{{{\boldsymbol{\phi }}}}}}}}\in {{\mathbb{R}}}^{1\times 60}$. **e** Discovered RD equations: evolution of the sparse coefficients ${{{{{{{{\boldsymbol{\lambda }}}}}}}}}^{u}\in {{\mathbb{R}}}^{110\times 1}$ and ${{{{{{{{\boldsymbol{\lambda }}}}}}}}}^{v}\in {{\mathbb{R}}}^{110\times 1}$ (**Λ** = [***λ***^*u*^***λ***^*v*^]) for 110 candidate functions ${{{{{{{\boldsymbol{\phi }}}}}}}}\in {{\mathbb{R}}}^{1\times 110}$ used to reconstruct the *u*-equation and the *v*-equation, respectively.

We observe the efficacy and robustness of our methodology on a group of canonical PDEs used to represent a wide range of physical systems with nonlinear, periodic and/or chaotic behaviors. In particular, we discover the closed forms of Burgers’, Kuramoto–Sivashinsky (KS), nonlinear Schrödinger, Navier–Stokes (NS), and *λ*-*ω* reaction–diffusion (RD) equations from scarce and noisy time-series measurements recorded by a number of sensors at fixed locations (data are polluted with Gaussian white noise) from a single I/BC. Results are presented in Table 1, Fig. 2 and Supplementary Note 2.1, which show quite accurate discovery and demonstrate satisfactory performance of the proposed method and its robustness to measurement data scarcity and noise. We also extensively compare our method with SINDy considering different levels of data scarcity and noise (summarized in Supplementary Note 2.2 and Supplementary Table 1).

**Table 1:** Summary of the PINN-SR discovery results in the context of accuracy for a range of canonical models.

PDE name	Err. (N-0%)	Err. (N-1%)	Err. (N-10%)	Description of data discretization
Burgers’	0.01 ± 0.01%	0.19 ± 0.11%	0.88 ± 0.03%	$x\in {[-8,8]}_{\tilde{n} = 256}$, $t\in {[0,10]}_{\tilde{n} = 101}$, sub. 3.19%
KS	0.07 ± 0.01%	0.61 ± 0.04%	0.94 ± 0.05%	$x\in {[0,100]}_{\tilde{n} = 1024}$, $t\in {[0,100]}_{\tilde{n} = 251}$, sub. 12.6%%
Schrödinger	0.09 ± 0.04%	0.65 ± 0.29%	0.08 ± 0.03%	$x\in {[-4.5,4.5]}_{\tilde{n} = 512}$, $t\in {[0,\pi ]}_{\tilde{n} = 501}$, sub. 37.5%
NS	0.66 ± 0.72%	0.86 ± 0.63%	1.22 ± 0.69%	$x\in {[0,9]}_{\tilde{n} = 449}$, $y\in {[-2,2]}_{\tilde{n} = 199}$, $t\in {[0,30]}_{\tilde{n} = 151}$, sub. 0.22%
*λ*-*ω* RD	0.07 ± 0.08%	0.25 ± 0.30%	1.84 ± 1.48%	$x,y\in {[-10,10]}_{\tilde{n} = 256}$, $t\in {[0,10]}_{\tilde{n} = 201}$, sub. 0.29%

The error is defined as the average relative error of the identified non-zero coefficients w.r.t. the ground truth. The percentage values in the parentheses denote the noise levels (e.g., noise free 0%, 1% and 10%) and the subscript $\tilde{n}$ represents the number of discretization. Our method is also compared with SINDy (the PDE-FIND approach presented in ref. ^6^) as illustrated in Supplementary Table 1. It is noted that much less measurement data polluted with a higher level of noise are used in our discovery. Gaussian white noise is added to the synthetic response with the noise level defined as the root-mean-square ratio between the noise and the exact solution.

*Burgers’ Equation:* We first consider a dissipative system with the dynamics governed by a 1D viscous Burgers’ equation expressed as *u*_*t*_ = −*uu*_*x*_ + *νu*_*xx*_, where *ν* (equal to 0.1) denotes the diffusion coefficient. The equation describes the decaying stationary viscous shock of a system after a finite period of time, commonly found in simplified fluid mechanics, nonlinear acoustics and gas dynamics. We test the PINN-SR approach on the recorded traveling shock waves from the solution to Burgers’ equation subjected to a Gaussian initial condition. In particular, 10 sensors are randomly placed at fixed locations among the 256 spatial grids and record the wave for 101 time steps, leading to 3.19% of the dataset used in ref. ^6^. A full description of the dataset, design of the library of candidate functions (16 terms) and model training is given in Supplementary Note 2.1.1. Figure 2a shows the discovered Burgers’ equation for a dataset with 10% noise. The evolution of the coefficients ${{{{{{{\mathbf{\Lambda }}}}}}}}\in {{\mathbb{R}}}^{16\times 1}$ illustrates robust convergence to the ground truth (error about 0.88%), resulting in accurate discovery. The trained PINN-SR properly reproduces the dynamical response from noisy measurements (e.g., the full-field *ℓ*_2_ prediction error is 1.32%) as shown in Supplementary Fig. 1. The ADO algorithm converges only after the first alternating iteration and shows capacity to recover the correct sparsity pattern of the PDE. We also discover the Burgers’ equation with an unknown/unmeasured source $\sin (x)\sin (t)$, given scarce *u*-measurement with 10% noise. When discovering the underlying governing equation, the source should be considered and reconstructed concurrently. In this case, we incorporate 14 source candidate functions, composed of $\{\sin (t),\sin (x),\cos (t),\cos (x)\}$ and their combination, into the aforementioned library, resulting in a total of 30 candidate terms for simultaneous discovery of the PDE and reconstruction of the unknown source. The corresponding discovery result is summarized in Supplementary Fig. 12, which includes the discovered equation and source function, the evolution of sparse coefficients ${{{{{{{\mathbf{\Lambda }}}}}}}}\in {{\mathbb{R}}}^{30\times 1}$, and the predicted full-field response. It turns out that both PDE and source terms along with their coefficients are well identified. Nevertheless, if the source is very complex with its general expression or form completely unknown, distinct challenges arise when designing the source candidate functions. This may require an extraordinarily large-space library to retain diversifying representations, and thus pose additional computational complexity for accurate discovery of the PDEs. More discussions are presented in Supplementary Note 3.1.

*Kuramoto–Sivashinsky (KS) Equation:* Another dissipative system with intrinsic instabilities is considered, governed by the 1D Kuramoto-Sivashinsky (KS) equation *u*_*t*_ = −*uu*_*x*_ − *u*_*xx*_ − *u*_*xxxx*_, where the reverse diffusion term −*u*_*xx*_ leads to the disruptive behavior while the fourth-order derivative *u*_*xxxx*_ introduces chaotic patterns as shown in Supplementary Fig. 2, making it an ideal test problem for equation discovery. The KS equation is widely used to model the instabilities in laminar flame fronts and dissipative trapped-ion modes among others. We randomly choose 320 points as fixed sensors and record the wave response for 101 time steps, resulting in 12.6% of the dataset used in ref. ^6^. A total of 36 candidate functions are employed to construct the underlying PDE. Detail description of this example is found in Supplementary Note 2.1.2. It is notable that the chaotic behavior poses significant challenges in approximating the full-field spatiotemporal derivatives, especially the high-order *u*_*xxxx*_, from poorly measured data for discovery of such a PDE. Existing methods (e.g., the family of SINDy methods^6,7^) eventually fail in this case given very coarse and noisy measurements. Nevertheless, PINN-SR successfully distils the closed form of the KS equation from subsampled sparse data with 10% noise, shown in Fig. 2b. The evolution of the coefficients ${{{{{{{\mathbf{\Lambda }}}}}}}}\in {{\mathbb{R}}}^{36\times 1}$ in Fig. 2b illustrates that both the candidate terms and the corresponding coefficients are correctly identified (close to the original parameters; error around 0.94%) within a few ADO iterations. The predicted full-field wave by the trained PINN-SR also coincides with the exact solution at a relative *ℓ*_2_ error of 2.14% (Supplementary Fig. 2).

*Nonlinear Schrödinger equation:* In the third example, we discover the nonlinear Schrödinger equation, *iu*_*t*_ = −0.5*u*_*xx*_ − ∣*u*∣^2^*u*, where *u* is a complex field variable. This well-known equation is widely used in modeling the propagation of light in nonlinear optical fibers, Bose-Einstein condensates, Langmuir waves in hot plasmas, and so on. We take 37.5% subsamples (e.g., randomly selected from the spatial grids) of the dataset as shown in Table 1 to construct the PDE using 40 candidate functions ${{{{{{{\boldsymbol{\phi }}}}}}}}\in {{\mathbb{R}}}^{1\times 40}$. Since the function is complex-valued, we model separately the real part (*u*_*R*_) and the imaginary part (*u*_*I*_) of the solution in the output of the DNN, assemble them to obtain the complex solution *u* = *u*_*R*_ + *iu*_*I*_, and construct the complex-valued candidate functions for PDE discovery. To avoid complex gradients in optimization, we use the modulus ∣*u*∣, instead of the *ℓ*_2_ norm shown in Eq. (5), for the residual physics loss ${{{{{{{{\mathcal{L}}}}}}}}}_{p}$ (see Supplementary Note 2.1.3 for more details). Figure 2c shows the discovered Schrödinger equation for the case of 10% noise. The evolution history of the sparse coefficients ${{{{{{{\mathbf{\Lambda }}}}}}}}\in {{\mathbb{R}}}^{40\times 1}$ clearly shows the convergence to the actual values (Fig. 2c; error about 0.08%) resulting in accurate closed-form identification of the PDE, while the reconstructed full-field response, for both real and imaginary parts, matches well the exact solution with a slight relative *ℓ*_2_ error of 0.26% (Supplementary Fig. 3).

*Navier-Stokes (NS) Equation:* We consider a 2D fluid flow passing a circular cylinder with the local rotation dynamics governed by the well-known Navier-Stokes vorticity equation *w*_*t*_ = − (**u** ⋅ ∇ )*w* + *ν*∇^2^*w*, where *w* is the spatiotemporally variant vorticity, **u** = {*u*, *v*} denotes the fluid velocities, and *ν* is the kinematic viscosity (*ν* = 0.01 at Reynolds number 100). We leverage the open simulation data^6^ and subsample a dataset of the flow response {*u*, *v*, *w*} at 500 spatial locations randomly picked within the indicated region in Supplementary Fig. S4, which record time series for 60 time steps. The resulting dataset is only 10% of that used in ref. ^6^. A comprehensive discussion of this example is found in Supplementary Note 2.1.4. Figure 2d summarizes the result of the discovered NS equation for a dataset with 10% noise. It is encouraging that the uncovered PDE expression is almost identical to the ground truth, for both the derivative terms and their coefficients, even under 10% noise corruption. The coefficients ${{{{{{{\mathbf{\Lambda }}}}}}}}\in {{\mathbb{R}}}^{60\times 1}$, corresponding to 60 candidate functions ${{{{{{{\boldsymbol{\phi }}}}}}}}\in {{\mathbb{R}}}^{1\times 60}$, converge very quickly to the correct values with precise sparsity right after the first ADO iteration (Fig. 2d). The vorticity patterns and magnitudes are also well predicted as indicated by the snapshot (at *t* = 23.8) shown in Supplementary Fig. 5 (the full-field *ℓ*_2_ error for all snapshots is about 2.58%). This example provides a compelling test case for the proposed PINN-SR approach which is capable of discovering the closed-form NS equation with scarce and noisy data.

*Reaction–diffusion (RD) equations:* The examples above are mostly low-dimensional models with limited complexity. We herein consider a *λ*-*ω* reaction–diffusion (RD) system in a 2D domain with the pattern forming behavior governed by two coupled PDEs: *u*_*t*_ = 0.1∇^2^*u* + *λ*(*g*)*u* − *ω*(*g*)*v* and *v*_*t*_ = 0.1∇^2^*v* + *ω*(*g*)*u* + *λ*(*g*)*v*, where *u* and *v* are the two field variables, *g* = *u*^2^ + *v*^2^, *ω* = − *g*^2^, and *λ* = 1 − *g*^2^. The RD equations exhibit a wide range of behaviors including wave-like phenomena and self-organized patterns found in chemical and biological systems. The particular RD equations considered here display spiral waves subjected to periodic boundary conditions. Full details on the dataset, selection of candidate functions and hyper-parameter setup of the PINN-SR model are given in Supplementary Note 2.1.5. Fig. 2e shows the evolution of the sparse coefficients ${{{{{{{{\boldsymbol{\lambda }}}}}}}}}^{u},{{{{{{{{\boldsymbol{\lambda }}}}}}}}}^{v}\in {{\mathbb{R}}}^{110\times 1}$ for 110 candidate functions ${{{{{{{\boldsymbol{\phi }}}}}}}}\in {{\mathbb{R}}}^{1\times 110}$, given a dataset with 10% noise. Both the sparse terms and the associated coefficients are precisely identified to form the the closed-form equations (as depicted in Fig. 2e). Due to the complexity of the PDEs and the high dimension, slightly more epochs are required in ADO to retain reliable convergence. The predicted response snapshots (e.g., at *t* = 2.95) by the trained PINN-SR in Supplementary Fig. 6 are close to the ground truth. This example shows especially the great ability and robustness of our method for discovering governing PDEs for high-dimensional systems from highly noisy data.

### Discovery of PDEs with multiple independent datasets

To demonstrate the “root-branch” network presented in Fig. 1b for the discovery of PDE(s) based on multiple independent datasets sampled under different I/BCs, we consider (1) the 1D Burgers’ equation with light viscosity that exhibits a shock behavior, and (2) a 2D Fitzhugh–Nagumo (FN) type reaction–diffusion system that describes activator-inhibitor neuron activities excited by external stimulus. The measurement data are sparsely sampled (e.g., time series or snapshots) with 10% noise under three different I/BCs. Note that the I/BCs are unnecessarily either measured or known a priori since the measurements already reflect the specific I/BC which holds uniquely one-to-one mapping to the system response. The discovery results are discussed as follows.

**Fig. 3:**
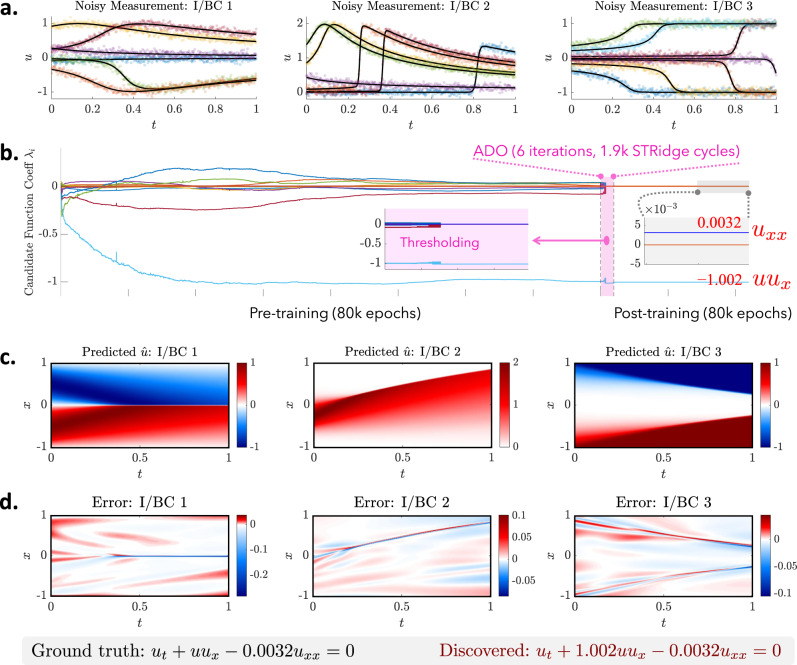
Discovered Burgers’ equation with small viscosity based on datasets sampled under three I/BCs with 10% noise. **a** Visualization of noisy measurements for the three datasets. Note that there are 30 sensors and only a few are illustrated in this figure. **b** Evolution of the sparse coefficients ${{{{{{{\mathbf{\Lambda }}}}}}}}\in {{\mathbb{R}}}^{16\times 1}$ for 16 candidate functions ${{{{{{{\boldsymbol{\phi }}}}}}}}\in {{\mathbb{R}}}^{1\times 16}$ used to construct the PDE, where the color represents the coefficient value. The correct terms (*uu*_*x*_ and *u*_*xx*_) and their coefficients are successfully identified while other redundant terms are eliminated by ADO. **c**, **d** The predicted responses and errors for three I/BC cases. The ground truth is not listed herein since the visualization is almost indistinguishable from the prediction (see Supplementary Fig. 7). The relative full-field *ℓ*_2_ error of the stacked prediction is 0.65%.

*Burgers’ equation with shock behavior:* In this example, we test the previously discussed Burgers’ equation with a small diffusion/viscosity parameter (*ν* = 0.01/*π* ≈ 0.0032) based on datasets generated by imposing three different I/BCs. Such a small coefficient creates shock formation in a compact area with sharp gradient (see Fig. 3c) that could challenge the DNN’s approximation ability and thus affect the discovery. The three initial and Dirichlet boundary conditions include

$$	\,{{\mbox{I/BC 1:}}}\,u(x,0) =-\!\sin (\pi x),u(-1,t)=u(1,t)=0\\ 	 \,{{\mbox{I/BC 2:}}}\,u(x,0) ={{{{{{{\mathcal{G}}}}}}}}(x),u(-1,t)=u(1,t)=0\\ 	 \,{{\mbox{I/BC 3:}}}\,u(x,0) =-{x}^{3},u(-1,t)=1,u(1,t)=-1$$

where ${{{{{{{\mathcal{G}}}}}}}}$ denotes a Gaussian function. Although the measurement datasets for different I/BCs exhibit completely distinct system responses, they obey the same underlying PDE, namely, *u*_*t*_ = − *uu*_*x*_ + 0.0032*u*_*xx*_. For all I/BCs, we assume that there are 30 sensors randomly deployed in space (*x* ∈ [−1, 1]) measuring the wave traveling (e.g., *u*) for 500 time instants (*t* ∈ [0, 1]). A denser sensor grid is needed herein, compared with the previous Burgers’ example, in order to capture the shock behaviors. Figure 3a shows some of the measurements recorded by six typical sensors under 10% noise. A three-branch network (*r* = 3) shown in Fig. 1b is used for discovery. The full description of the dataset, the library of candidate functions (16 terms) and model training is given in Supplementary Note 2.3.1. Figure 3b depicts the evolution of the coefficients (${{{{{{{\mathbf{\Lambda }}}}}}}}\in {{\mathbb{R}}}^{16\times 1}$) of candidate functions, where the correct terms in the library (*uu*_*x*_ and *u*_*xx*_) are successfully distilled while other redundant terms are eliminated (e.g., hardly thresholded to zero) by ADO. The coefficients of the active terms are accurately identified as well (in particular the small viscosity parameter that leads to shock formation, e.g., 0.0039). The discovered PDE reads *u*_*t*_ = −1.002*uu*_*x*_ + 0.0032*u*_*xx*_. Figure 3c, d shows the predicted responses and errors for three I/BC cases, with a stacked full-field *ℓ*_2_ error of 0.65%.

**Fig. 4:**
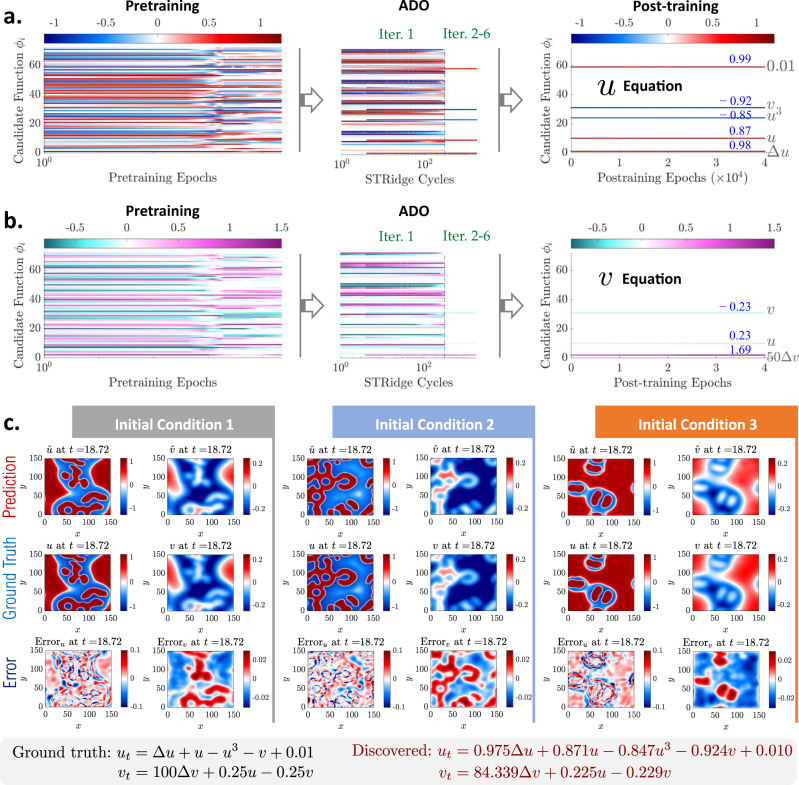
Discovered Fitzhugh–Nagumo equations based on data sampled under three initial conditions (ICs) with 10% noise. **a** Evolution of the sparse coefficients ${{{{{{{{\boldsymbol{\lambda }}}}}}}}}_{u}\in {{\mathbb{R}}}^{72\times 1}$ for 72 candidate functions used to construct the first PDE (*u*-equation), where the color represents the coefficient value. **b** Evolution of the sparse coefficients ${{{{{{{{\boldsymbol{\lambda }}}}}}}}}_{v}\in {{\mathbb{R}}}^{72\times 1}$ for the second PDE (*v*-equation). For visualization purpose, we re-scale the identified coefficients of the constant stimulus term “1” in the *u*-equation by multiplying 100 and of the diffusion term Δ*v* in the *v*-equation by dividing 50. **c** Snapshots of predicted response, ground truth and error distributions for all three ICs at an unmeasured time instance (*t* = 18.72). The relative *ℓ*_2_ error for the predicted full-field response (stacked *u* and *v*) is 5.02%.

*FitzHugh–Nagumo (FN) reaction–diffusion system:* We consider the FitzHugh–Nagumo (FN) type reaction–diffusion system, in a 2D domain Ω = [0, 150] × [0, 150] with periodic boundary conditions, whose governing equations are expressed by two coupled PDEs: *u*_*t*_ = *γ*_*u*_Δ*u* + *u* − *u*^3^ − *v* + *α* and *v*_*t*_ = *γ*_*v*_Δ*v* + *β*(*u* − *v*). Here, *u* and *v* represent two interactive components/matters (e.g., biological), *γ*_*u*_ = 1 and *γ*_*v*_ = 100 are diffusion coefficients, *α* = 0.01 and *β* = 0.25 are the coefficients for reaction terms, and Δ is the Laplacian operator. The FN equations are commonly used to describe biological neuron activities excited by external stimulus (*α*), which exhibit an activator-inhibitor system because one equation boosts the production of both components while the other equation dissipates their new growth. Three random fields are taken as initial conditions to generate three independent datasets for discovery, each of which consists of 31 low-resolution snapshots (projected into a 31 × 31 grid) down-sampled from the high-fidelity simulation under a 10% noise condition (see Supplementary Fig. 8). We assume the diffusion terms (Δ*u* and Δ*v*) are known in the PDEs, whose coefficients (*γ*_*u*_ and *γ*_*v*_) yet need to be identified. A library with 72 candidate functions (${{{{{{{\boldsymbol{\phi }}}}}}}}\in {{\mathbb{R}}}^{1\!\times \!72}$) is designed for discovery of the coupled PDEs (in particular, the nonlinear reaction terms). Similar to the previous example, a root-branch network shown in Fig. 1b is employed for discovery. More description of the data generation, the specific candidate functions and model training can be found in Supplementary Note 2.3.2. Figure 4a, b depicts the evolution of the sparse coefficients ${{{{{{{{\boldsymbol{\lambda }}}}}}}}}^{u},{{{{{{{{\boldsymbol{\lambda }}}}}}}}}^{v}\in {{\mathbb{R}}}^{72\times 1}$ for 72 candidate functions. The pre-training step provides a redundant projection of the system onto 72 candidates; however, minor candidates are pruned out right after the first ADO iteration. The rest ADO iterations continue to refine all the trainable parameters including ***θ***, ***λ***^*u*^ and ***λ***^*v*^. The finally discovered PDEs are listed in Fig. 4 in comparison with the ground truth. It is seen that the form of the PDEs is precisely uncovered with all correct active terms (including the unknown external stimulus in the first equation). The corresponding identified coefficients are generally close to the ground truth except the diffusion coefficient for *v* (i.e., *γ*_*v*_) which seems to be a less sensitive parameter according to our test. It should be noted that, given very scarce and noisy measurement datasets in this example, the “root-branch” DNN is faced with challenges to accurately model the solutions with sharp propagating fronts (see Fig. 4c). The less accurate solution approximation by DNN then affects the discovery precision. This issue can be naturally alleviated by increasing the spatiotemporal measurement resolution (even still under fairly large noise pollution, e.g., 10%). Nevertheless, the exact form of the PDEs is successfully discovered in this challenging example, which is deemed more important since the coefficients can be further tuned/calibrated when additional data arrives. Figure 4c shows typical snapshots of the predicted *u* and *v* components, the ground truth reference and the error distributions for one unmeasured time instance (*t* = 18.72). The stacked full-field *ℓ*_2_ error is 5.02%.

### Experimental discovery of cell migration and proliferation

The last example is placed to demonstrate the proposed approach for discovering a governing PDE that describes cell migration and proliferation, based on the sparse and noisy experimental data collected from in vitro cell migration (scratch) assays^46^. The 1D cell density distributions at different time instants (0 h, 12 h, 24 h, 36 h, 48 h) were extracted from high-resolution imaging via image segmentation and cell counting. A series of assays were performed under different initial cell densities (e.g., the total number of cells spans from 10,000 to 20,000 following the designated initial distribution in the test well shown in Supplementary Fig. 10 at *t* = 0h). A more detailed description of the experiment setup and datasets can be found in ref. ^46^. Our objective herein is to uncover a parsimonious PDE for modeling the dynamics of cell density *ρ*(*x*, *t*). Here, we consider four scenarios with the initial number of cells ranging from 14,000, 16,000, 18,000 to 20,000. We take the mean of the test data from three identically-prepared experimental replicates for each scenario (see Supplementary Fig. 10) to train our model shown in Fig. 1a for PDE discovery. Given our prior knowledge that the cell dynamics can be described by a diffusion (migration) and reaction (proliferation) process, we assume the PDE holds the form of ${\rho }_{t}=\gamma {\rho }_{xx}+{{{{{{{\mathcal{F}}}}}}}}(\rho )$, where *γ* is the unknown diffusion coefficient and ${{{{{{{\mathcal{F}}}}}}}}$ denotes the underlying nonlinear reaction functional. We use 8 additional candidate terms (e.g., {1, *ρ*, *ρ*^2^, *ρ*^3^, *ρ*_*x*_, *ρρ*_*x*_, *ρ*^2^*ρ*_*x*_, *ρ*^3^*ρ*_*x*_}) to reconstruct ${{{{{{{\mathcal{F}}}}}}}}$, whose coefficients are sparse. Hence, the total number of trainable coefficients remains 9 (e.g., ${{{{{{{\mathbf{\Lambda }}}}}}}}\in {{\mathbb{R}}}^{9\times 1}$). We believe incorporating our domain-specific prior knowledge is reasonable and should be encouraged in interpretable model discovery, which could help improve our solution confidence when available data is very sparse and noisy (e.g., in this example). Other details on the PINN-SR model setting and training can be found in Supplementary Note 2.4.

**Fig. 5:**
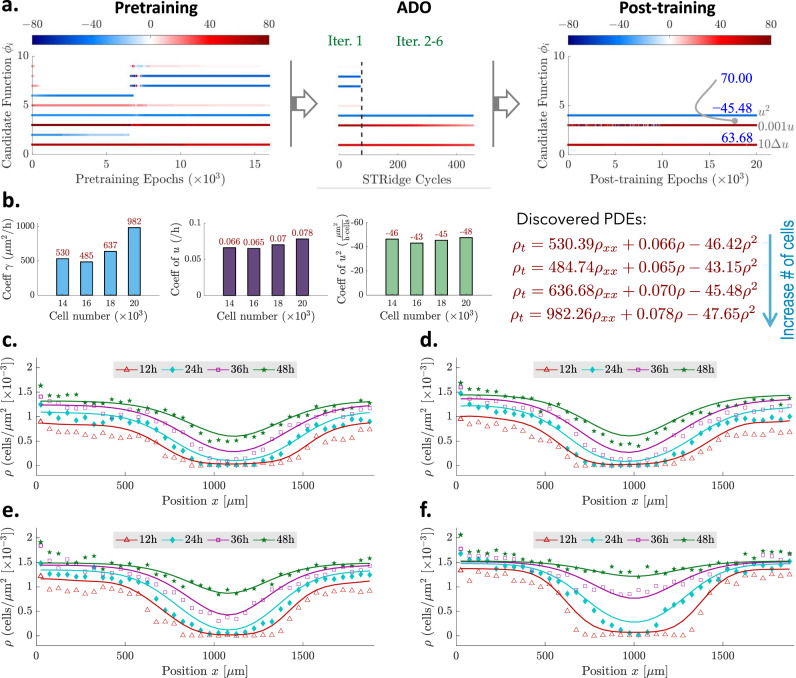
Discovery result for cell migration and proliferation. **a** Example evolution of the sparse coefficients ${{{{{{{\mathbf{\Lambda }}}}}}}}\in {{\mathbb{R}}}^{9\times 1}$ for 9 candidate functions used to construct the underlying PDE for the case of 18,000 cells. The diffusion and reaction coefficients for Δ*u* and *u* are re-scaled for visualization purpose. **b** Discovered active terms {Δ*ρ*, *ρ*, *ρ*^2^}, their coefficients and the corresponding PDEs for 14,000, 16,000, 18,000 and 20,000 cells, respectively. **c**, **f** Simulated cell densities at different time instants based on the discovered PDEs for 14,000, 16,000, 18,000 and 20,000 cells, respectively, where the measurement at 0h is used as the initial condition while *ρ*_*x*_(*x* = 0, *t*) = *ρ*_*x*_(*x* = 1900, *t*) = 0 is employed as the Neumann boundary condition. The simulation result is represented by solid curves while the markers denote the measurement data.

Figure 5a shows the evolution of 9 coefficients for the example case of 18,000 cells, where redundant candidate terms are pruned right after the first ADO iteration via hard thresholding of the corresponding coefficients to zero. The next ADO iterations followed by post-tuning refine the coefficients of active terms for final reconstruction of the PDE. Figure 5b depicts the identified active term coefficients and the corresponding PDEs for different quantities of cells, sharing a unified form of *ρ*_*t*_ = *γρ*_*xx*_ + *λ*_1_*ρ* + *λ*_2_*ρ*^2^ which exactly matches the famous Fisher-Kolmogorov model^47^. The rates of migration (diffusion) and proliferation (reaction) generally increase along with the number of cells, as seen from the identified coefficients in Fig. 5b. With the discovered PDEs, we simulate/predict the evolution of cell densities at different time instants (12h, 24h, 36h and 48h) presented in Fig. 5c–f, where the measurement at 0h is used as the initial condition while *ρ*_*x*_(*x* = 0, *t*) = *ρ*_*x*_(*x* = 1900, *t*) = 0 is employed as the Neumann boundary condition. The satisfactory agreement between the prediction and the measurement provides a clear validation of our discovered PDEs. It is noted that the extremely scarce and noisy experimental datasets unfortunately pose intractable challenge for any other existing methods (e.g., SINDy^5,6^) to produce a reasonable discovery. This experimental example further demonstrates the strength and capacity of the proposed methodology in regard to handling high level of data scarcity and noise for PDE discovery.

## Discussion

In summary, we have presented a novel deep learning method for discovering physical laws, in particular parsimonious closed-form PDE(s), from scarce and noisy data (commonly seen in scientific investigations and real-world applications) for multi-dimensional nonlinear spatiotemporal systems. This approach combines the strengths of DNNs for rich representation learning of nonlinear functions, automatic differentiation for accurate derivative calculation as well as *ℓ*_0_ sparse regression to tackle the fundamental limitation faced by existing sparsity-promoting methods that scale poorly with respect to data noise and scarcity. The use of collocation points (having no correlation with the measurement data) can render the proposed framework tolerable to scarce and noisy measurements, making the DNN for PDE solution approximation generalizable (see Supplementary Note 3.3). The special network architecture design is able to account for multiple independent datasets sampled under different initial/boundary conditions. An alternating direction optimization strategy is proposed to simultaneously train the DNN and determine the optimal sparse coefficients of selected candidate terms for reconstructing the PDE(s). The synergy of DNN and sparse PDE representation results in the following outcome: the DNN provides accurate modeling of the solution and its derivatives as a basis for constructing the governing equation(s), while the sparsely represented PDE(s) in turn informs and constraints the DNN which makes it generalizable and further enhances the discovery. The overall approach is rooted in a comprehensive integration of bottom-up (data-driven) and top-down (physics-informed) processes for scientific discovery, with fusion of physics-informed deep learning, sparse regression and optimization. We demonstrate this method on a number of dynamical systems exhibiting nonlinear spatiotemporal behaviors (e.g., chaotic, shock, propagating front, etc.) governed by multi-dimensional PDEs based on either single or multiple datasets, numerically or experimentally. Results highlight that the approach is capable of accurately discovering the exact form of the governing equation(s), even in an information-poor space where the multi-dimensional measurements are scarce and noisy. The proposed method also maintains satisfactory robustness against different types of noises (Gaussian and non-Gaussian; see Supplementary Note 3.4) for PDE discovery.

There still remain some potential limitations associated with the present PINN-SR framework for physical law discovery. Firstly, we have to admit that the computational cost of PINN-SR is much higher compared with the state-of-the-art SINDy method, primarily due to the time-consuming DNN training (see Supplementary Note 2.2). However, the critical bottleneck of SINDy lies in its requirement of large high-quality (clean) structured measurement data, owing to its use of numerical differentiation, which poses critical limitation of SINDy in practical applications where data is sparse and noisy (e.g., the experimental data in the cell migration and proliferation example). There is obviously a trade-off between computational efficiency and need of high-quality data. Another limitation is that, although the fully connected DNN used in this work has advantage of analytical approximation of the PDE derivatives via automatic differentiation, directly applying it to model the solution of higher dimensional systems (such as long/short-term response evolution in a 3D domain) results in computational bottleneck and optimization challenges, e.g., due to the need for a vast number of collocation points to maintain satisfactory accuracy. Advances in discrete DNNs with spatiotemporal discretization (e.g., the convolutional long short-term memory network (ConvLSTM)^48^ or similar) have the potential to help resolve this challenge, which will be demonstrated in our future work. In addition, the “root-branch” scheme might suffer from scalability issues when a large number of independent datasets sampled under various I/BCs are available, resulting in many branches of the network for PDE solution approximation. The number of DNN trainable variables, the requirement of collocation points for retaining solution accuracy, and thus the computing memory, will grow in general linearly with the number of independent datasets (e.g., ${{{{{{{\mathcal{O}}}}}}}}(r)$). Nevertheless, this issue can be potentially well resolved by multi-GPU parallelization. Ideally, if the I/BCs are known a priori and can be parameterized under the condition that large and diverse datasets are available, a parametric DNN learning scheme^39^ or neural operator learning^49,50^ could be developed into the proposed PINN-SR for parametric PDE solution approximation that accounts for different I/BCs. Nevertheless, we emphasize that the assumption of large datasets is out of the scope of our present study, since this requirement is generally hard to meet in equation discovery related applications where data is commonly scarce.

The current version of PINN-SR is inapplicable to the scenario where the PDE coefficients are variant (e.g., time and/or space dependent). However, given PINN’s ability of identifying varying coefficients of PDEs^51^, PINN-SR can be naturally extended to discover the closed form of PDEs where the varying coefficients are separately modeled and identified. Moreover, PINN is not good at modeling system with chaotic behaviors or sharp propagating wave fronts, primarily due to the way of its solution field approximation with global basis. This limitation is particularly evident when the labeled data is missing (e.g., solving PDEs given I/BCs^52^) or when the model form is unknown (e.g., data-driven modeling with constrained by hidden physics^43^). However, such a limitation can be apparently alleviated, when the labeled data is relatively rich and a clear PDE model is explicitly given (e.g., the library-based model). Nevertheless, the learned full-field response still possesses errors in the propagating wave fronts if the training data is sparse and noisy. Although these errors did not affect much the discovered PDE structure, they result in less accurate identification of PDE coefficients. A network with local basis support might help resolve this issue. Lastly, while PINN-SR relies on a pre-defined library of candidate terms, designing a priori inclusive but not unnecessarily large library remains a difficult task (see more details in Methods). Combining expression trees^53^ or symbolic neural networks^54^ with PINN and automatic differentiation has the potential to break the limitation of library-based methods for PDE discovery under sparse and noisy data conditions.

Several other aspects (including optimal placement of sensors, convergence history, parametric study on the network size, list of hyper-parameters used in the examples, and other limitations of the method) are further discussed in Supplementary Note 3.5–3.9.

## Methods

The innovations of this work are built upon seamless integration of the strengths of deep neural networks for rich representation learning, physics embedding, automatic differentiation and sparse regression to (1) approximate the solution of system variables, (2) compute essential derivatives, as well as (3) identify the key derivative terms and parameters that form the structure and explicit expression of the PDE(s). The technical contributions include: (1) a “root-branch” network, constrained by unified underlying physics, that is capable of dealing with a small number of multi-datasets coming from different I/BCs, and (2) a simple, yet effective, multi-step training strategy for optimization of heterogeneous parameters. The resulting approach is able to deal with scarce/sparse and highly noisy measurement data while accounting for different initial/boundary conditions. The key method components are discussed below.

### Network architecture

The proposed network architectures of PINN-SR are shown in Figs. 1a, b that respectively deal with single-I/BC dataset and multiple-I/BC (*r*) independent datasets. The latent solution **u** is interpreted by a dense (fully connected) DNN shown in Fig. 1a, namely, **u**^*θ*^ = **u**(**x**, *t*; ***θ***), for the case of single dataset, while a “root-branch” dense DNN depicted in Fig. 1b is designed to approximate the latent solutions **u**_*i*_ (*i* = 1, …, *r*) corresponding to different I/BCs, viz., ${{{{{{{{\bf{u}}}}}}}}}_{i}^{\theta }={{{{{{{\bf{u}}}}}}}}\left({{{{{{{\bf{x}}}}}}}},t;{{{{{{{{\boldsymbol{\theta }}}}}}}}}^{(0)},{{{{{{{{\boldsymbol{\theta }}}}}}}}}^{(i)}\right)$, for multiple independent datasets. Here, ***θ***’s denote the DNN trainable parameters. The DNNs take the spatiotemporal domain coordinates {**x**, *t*} as input followed by multiple fully connected feedforward hidden layers (each layer has dozens of nodes). We use the hyperbolic tangent (tanh) or sine (sin) as the universal activation function thanks to their strength for high-order differentiation and unbiased estimation for both positive and negative values. The sin function is used when the system response exhibits periodic patterns. The output later is based on linear activation for universal magnitude mapping. When multiple datasets are available, e.g. sampled from different I/BCs, domain coordinates are input to the “root” net (shared hidden layers), followed by *r* “branch” nets (individual hidden layers) that predict system response corresponding to each I/BC or dataset. The “root” learns the common patterns across all datasets (e.g., the homogeneous part of the solution) while the “branches” learn specific details determined by each I/BC for each independent dataset (e.g., the causality attributed by a specific I/BC). The resulting “root-branch” network, constrained by unified underlying physics, is capable of accounting for different I/BCs. Such an architecture integrates information from different measurements at the expense of larger computational efforts and produces solution approximations satisfying a unified physics (e.g., governing PDE(s)), which essentially strengthens PINN to perform multi-source data-driven modeling. The DNNs essentially play a role as a nonlinear functional to approximate the latent solution.

The DNN is connected to the physical law (reconstruction of PDE(s)) through a automatic differentiator where derivatives on **u**’s are evaluated at machine precision. The library of candidate functions ***ϕ***^*θ*^ can be computed from the DNNs. For the case of multiple independent datasets, the libraries ***ϕ***^(*i*)^ resulted from the “branch” nets are concatenated to build one unified ***ϕ***^*θ*^. If there is unknown source input, the candidate functions for **p** can also be incorporated into the library for discovery. The sparse representation of the reconstructed PDE(s) is then expressed in a residual form: ${{{{{{{{\bf{{{{{{{{\mathcal{R}}}}}}}}}}}}}}}}}^{\theta }:= {{{{{{{{\bf{u}}}}}}}}}_{t}^{\theta }-{{{{{{{{\boldsymbol{\phi }}}}}}}}}^{\theta }{{{{{{{\mathbf{\Lambda }}}}}}}}\to {{{{{{{\bf{0}}}}}}}}\,{{\mbox{s.t.}}}\,{{{{{{{\mathbf{\Lambda }}}}}}}}\in {{{{{{{\mathcal{S}}}}}}}}$, where ${{{{{{{{\bf{{{{{{{{\mathcal{R}}}}}}}}}}}}}}}}}^{\theta }\in {{\mathbb{R}}}^{1\times n}$ denotes the PDE residuals, ${{{{{{{\mathcal{S}}}}}}}}$ represents the sparsity constraint set, and *n* is the dimension of the system variable (e.g., ${{{{{{{\bf{u}}}}}}}}\in {{\mathbb{R}}}^{1\times n}$). Thus, the overall network architecture consists of heterogeneous trainable variables, namely, DNN parameters ${{{{{{{\boldsymbol{\theta }}}}}}}}\in {{\mathbb{R}}}^{{n}_{\theta }\times 1}$ and PDE coefficients ${{{{{{{\mathbf{\Lambda }}}}}}}}\in {{{{{{{\mathcal{S}}}}}}}}\subset {{\mathbb{R}}}^{s\times n}$, where *n*_*θ*_ denotes the number of DNN trainable parameters and *n*_*θ*_ ≫ *sn*.

### Physics-constrained sparsity-regularized loss function

The physics-constrained sparsity-regularized loss function, expressed in Eq. (3), is composed of three components, the data loss ${{{{{{{{\mathcal{L}}}}}}}}}_{d}$, the residual physics loss ${{{{{{{{\mathcal{L}}}}}}}}}_{p}$ and a sparsity regularization term imposed on **Λ**. The data loss function reads

4 $${{{{{{{{\mathcal{L}}}}}}}}}_{d}({{{{{{{\boldsymbol{\theta }}}}}}}};{{{{{{{{\mathcal{D}}}}}}}}}_{u})=\frac{1}{{N}_{m}}{\left\Vert {{{{{{{{\bf{u}}}}}}}}}^{\theta }-{{{{{{{{\bf{u}}}}}}}}}^{m}\right\Vert }_{2}^{2}$$

where **u**^*m*^ is the measurement data, **u**^*θ*^ is the corresponding DNN-approximated solution, *N*_*m*_ is the total number of data points, and ∥ ⋅ ∥_2_ denotes the Frobenius norm. The responses are stacked when multiple datasets are available, e.g., ${{{{{{{{\bf{u}}}}}}}}}^{m}=\{{{{{{{{{\bf{u}}}}}}}}}_{1}^{m},\ldots ,{{{{{{{{\bf{u}}}}}}}}}_{r}^{m}\}$ and ${{{{{{{{\bf{u}}}}}}}}}^{\theta }=\{{{{{{{{{\bf{u}}}}}}}}}_{1}^{\theta },\ldots ,{{{{{{{{\bf{u}}}}}}}}}_{r}^{\theta }\}$, where *r* ≥ 2, as shown in Fig. 1b. The PDE residuals ${{{{{{{{\bf{{{{{{{{\mathcal{R}}}}}}}}}}}}}}}}}^{\theta }$ are evaluated on a large number of randomly sampled collocation points ${{{{{{{{\mathcal{D}}}}}}}}}_{c}$, and used to form the residual physics loss function given by

5 $${{{{{{{{\mathcal{L}}}}}}}}}_{p}({{{{{{{\boldsymbol{\theta }}}}}}}},{{{{{{{\mathbf{\Lambda }}}}}}}};{{{{{{{{\mathcal{D}}}}}}}}}_{c})=\frac{1}{{N}_{c}}{\left\Vert \dot{{{{{{{{\bf{U}}}}}}}}}({{{{{{{\boldsymbol{\theta }}}}}}}})-{{{{{{{\mathbf{\Phi }}}}}}}}({{{{{{{\boldsymbol{\theta }}}}}}}}){{{{{{{\mathbf{\Lambda }}}}}}}}\right\Vert }_{2}^{2}$$

where $\dot{{{{{{{{\bf{U}}}}}}}}}$ and **Φ** denote respectively the discretization of the first-order time derivative term and the library of candidate functions evaluated on the collocation points; *N*_*c*_ is the total number of spatiotemporal collocation points. For the case of multiple datasets, $\dot{{{{{{{{\bf{U}}}}}}}}}$ and **Φ** are concatenated over the index of different I/BCs to ensure the identical physical law (in particular, the governing PDE(s)) is imposed, as depicted in Fig. 1b. Note that ${{{{{{{{\mathcal{L}}}}}}}}}_{d}$ ensures that the DNN accurately interpret the latent solution of the PDE(s) via fitting the data, while ${{{{{{{{\mathcal{L}}}}}}}}}_{p}$ generalizes and provides constraints for the DNN through reconstructing the closed form of the PDE(s). The *ℓ*_0_ regularization term in Eq. (3) promotes the sparsity of the coefficients **Λ** for sparse representation of the PDE(s).

### Alternating direction optimization

A brute-force training of the network via solving the optimization problem defined in Eq. (3) is highly intractable since the *ℓ*_0_ regularization makes this problem *np*-hard. Though relaxation of the *ℓ*_0_ term by the less rigorous *ℓ*_1_ regularization improves the well-posedness and enables the optimization in a continuous space, false-positive identification occurs where accurate sparsity of the PDE coefficients cannot be realized^44,45^. To address this challenge, we present an alternating direction optimization (ADO) algorithm that divides the overall optimization problem into a set of tractable subproblems to sequentially optimize ***θ*** and **Λ** within a few alternating iterations (denoted by *k*), namely,

6a $${{{{{{\mathbf{\Lambda }}}}}}}_{k+1}^{\star }:=\mathop{{{{{{\mathrm{arg}}}}}} \kern2pt {{{{{\mathrm{min}}}}}}}_{{{{{{\mathbf{\Lambda }}}}}}}[{\Vert \mathop{{{{{{\bf{U}}}}}}}\limits^{.}({{{{{{\boldsymbol{\theta }}}}}}}_{k}^{\star })-{{{{{\mathbf{\Phi }}}}}}({{{{{{\boldsymbol{\theta }}}}}}}_{k}^{\star }){{{{{\mathbf{\Lambda }}}}}}\Vert }_{2}^{2}+\beta \Vert {{{{{\mathbf{\Lambda }}}}}}{\Vert }_{0}]$$

6b $${{{{{{\boldsymbol{\theta }}}}}}}_{k+1}^{\star }:=\mathop{{{{{{\mathrm{arg}}}}}} \kern2pt {{{{{\mathrm{min}}}}}} }_{{{{{{\boldsymbol{\theta }}}}}}}[{ {\mathcal L} }_{d}({{{{{\boldsymbol{\theta }}}}}};{{{{{{\mathscr{D}}}}}}}_{u})+\alpha { {\mathcal L} }_{p}({{{{{\boldsymbol{\theta }}}}}},{{{{{{\mathbf{\Lambda }}}}}}}_{k+1}^{\star };{{{{{{\mathscr{D}}}}}}}_{c})]$$

The fundamental concept of the ADO algorithm is similar to (or can be regarded as a simplified version of) the alternating direction methods of multipliers^55^. In each alternating iteration *k* + 1, the sparse PDE coefficients **Λ** in Eq. (6a) are updated (denoted by ${{{{{{{{\mathbf{\Lambda }}}}}}}}}_{k+1}^{\star }$) via STRidge (a sequential thresholding regression process that serves as a proxy for *ℓ*_0_ regularization^5,6^), based on the DNN parameters from the previous iteration (e.g., ${{{{{{{{\boldsymbol{\theta }}}}}}}}}_{k}^{\star }$). The convergence analysis of STRidge can be found in ref. ^28^. The DNN parameters ***θ*** in the current iteration are then updated (denoted by ${{{{{{{{\boldsymbol{\theta }}}}}}}}}_{k+1}^{\star }$) through a standard DNN training algorithm (in particular, the combined Adam^56^ + L-BFGS^57^ optimizer), taking ${{{{{{{{\mathbf{\Lambda }}}}}}}}}_{k+1}^{\star }$ as known. Note that a sufficient number of epochs should be used when training the network in order to achieve satisfactory solution accuracy of ${{{{{{{{\boldsymbol{\theta }}}}}}}}}_{k+1}^{\star }$. The alternations between the sub-optimal solutions will lead to a high-quality optimization solution satisfying global convergence. The ADO sequence converges *q*-linearly (see Theorem 1 below), where *q* stands for “quotient”. Detailed theoretical analysis of generalized alternating optimization can be found in ref. ^58^. It is noteworthy that the Adam optimizer plays a role for global search while the L-BFGS optimizer takes responsibility of fine tuning in a local solution region. The learning rate of Adam ranges from 10^−5^ to 10^−3^ in the test examples. The algorithm design of ADO as well as the implementation details and specifications are given in Supplementary Algorithm 1, Algorithm 2 and Note 1.1.

#### Theorem 1

Let **Θ**^⋆^ = {***θ***^⋆^, **Λ**^⋆^} be a local minimizer of the total loss function ${{{{{{{\mathcal{L}}}}}}}}({{{{{{{\boldsymbol{\theta }}}}}}}},{{{{{{{\mathbf{\Lambda }}}}}}}};{{{{{{{{\mathcal{D}}}}}}}}}_{u},{{{{{{{{\mathcal{D}}}}}}}}}_{c}):{{\mathbb{R}}}^{\eta }\,\mapsto\, {\mathbb{R}}$ and let ${{{{{{{\mathcal{L}}}}}}}}$ be strictly convex in a neighborhood ${\mathfrak{N}}({{{{{{{{\mathbf{\Theta }}}}}}}}}^{\star },{{{{{{{\boldsymbol{\delta }}}}}}}})$, where *η* denotes the number of trainable parameters. We choose **0** < ***ϵ*** ≤ ***δ*** so that ${{{{{{{\mathcal{L}}}}}}}}$ is strictly convex on ${\mathfrak{N}}({{{{{{{{\mathbf{\Theta }}}}}}}}}^{\star },{{{{{{{\boldsymbol{\epsilon }}}}}}}})$. If ${{{{{{{\bf{y}}}}}}}}=\{{{{{{{{\boldsymbol{\theta }}}}}}}},{{{{{{{{\mathbf{\Lambda }}}}}}}}}^{\star }\}\in {\mathfrak{N}}({{{{{{{{\mathbf{\Theta }}}}}}}}}^{\star },\,{{{{{{{\boldsymbol{\epsilon }}}}}}}})$ and θ^*^ locally minimizes ${{{{{{{\mathcal{L}}}}}}}}({{{{{{{\boldsymbol{\theta }}}}}}}},{{{{{{{{\mathbf{\Lambda }}}}}}}}}^{\star };{{{{{{{{\mathcal{D}}}}}}}}}_{u},{{{{{{{{\mathcal{D}}}}}}}}}_{c})$, then θ^*^ is the unique global minimizer. This is also applicable to Λ^*^. For any admissible initial solution ${{{{{{{{\mathbf{\Theta }}}}}}}}}_{0}\in {\mathfrak{N}}({{{{{{{{\mathbf{\Theta }}}}}}}}}^{\star },{{{{{{{\boldsymbol{\epsilon }}}}}}}})$, the corresponding ADO iteration sequence converges to **Θ**^⋆^*q*-linearly in theory. The actual convergence rate depends on the error propagation in each ADO iteration.

Pre-training of PINN-SR is conducted before running the ADO algorithm for discovery, by simply replacing ∥**Λ**∥_0_ in Eq. (3) with ∥**Λ**∥_1_ where brute-force gradient-based optimization (e.g., Adam + L-BFGS) for both ***θ*** and **Λ** becomes applicable, namely,

7 $$\{{{{{{{\boldsymbol{\theta }}}}}}}^{\star },{{{{{{\mathbf{\Lambda }}}}}}}^{\star }\}=\mathop{{{{{{\mathrm{arg}}}}}}\, {{{{{\mathrm{min}}}}}} }_{\{{{{{{\boldsymbol{\theta }}}}}},{{{{{\mathbf{\Lambda }}}}}}\}}\{{ {\mathcal L} }_{d}({{{{{\boldsymbol{\theta }}}}}};{{{{{{\mathscr{D}}}}}}}_{u})+\alpha { {\mathcal {L}} }_{p}({{{{{\boldsymbol{\theta }}}}}},{{{{{\mathbf{\Lambda }}}}}};{{{{{{\mathscr{D}}}}}}}_{c})+\gamma \Vert {{{{{\mathbf{\Lambda }}}}}}{\Vert }_{1}\}$$

where *γ* denotes the *ℓ*_1_ regularization parameter. The *ℓ*_1_-regularized pre-training can accelerate the convergence of ADO by providing an admissible “initial guess”. During pre-training, the DNN learns the physics patterns underlying the sparse and noisy data, weakly constrained by the regression formulation of governing PDEs. Post-training (or post-tuning) is also applicable, which can be applied after the closed form (structure) of the PDE(s) is uncovered. This can be done by training the DNN along with the identification of the discovered non-zero coefficients, viz.,

8 $$\{{{{{{{\boldsymbol{\theta }}}}}}}^{\star },{{{{{{\mathbf{\Lambda }}}}}}}^{\star }\}=\mathop{{{{{{\mathrm{arg}}}}}}\, {{{{{\mathrm{min}}}}}} }_{\{{{{{{\boldsymbol{\theta }}}}}},{{{{{\mathbf{\Lambda }}}}}}\}}\{{ {\mathcal L} }_{d}({{{{{\boldsymbol{\theta }}}}}};{{{{{{\mathscr{D}}}}}}}_{u})+\alpha { {\mathcal {L}} }_{p}({{{{{\boldsymbol{\theta }}}}}},{{{{{\mathbf{\Lambda }}}}}};{{{{{{\mathscr{D}}}}}}}_{c})\}$$

where the initialization of the unknown parameters {***θ***, **Λ**} can be inherited from the ADO result. The post-training step is completely optional since the ADO method can already provide a high-quality solution as shown in the test examples. Nevertheless, the post-training could add additional discovery accuracy through fine tuning.

It is worthwhile to mention that the underlying intuition of multi-step training has been widely used and justified effective in the deep learning community, in particular, for DNN compression^59,60^ (e.g., network pre-training, weights pruning, and post-training). The proposed training strategy is similar to this commonly used procedure. The heuristic justification of the proposed 3-step training strategy reads: the pre-training phyase learns a good PDE solution approximator, ADO uncovers the parsimonious PDE structure, while the post-training stage fine-tunes the coefficients of the discovered PDE structure.

### Selection of hyper-parameters

A proper selection of hyper-parameters (e.g., *α*, *β*, *γ* and those required by Supplementary Algorithm 1 and Algorithm 2) guarantees the success of the proposed method for PDE discovery. In this study, the hyper-parameters are selected following the heuristically consistent criteria below.

- ${\alpha }$: This hyper-parameter balances the loss contributions from data and physics regularization for network training, which can be generally estimated based on the scale ratio between the measured response **u**^*m*^ and its temporal derivative ${{{{{{{{\bf{u}}}}}}}}}_{t}^{m}$ (estimated/approximated by finite difference). In particular, the magnitude of *α* is set to be similar to the deviation ratio between **u**^*m*^ and ${{{{{{{{\bf{u}}}}}}}}}_{t}^{m}$, namely, $\alpha \sim {r}_{\sigma }={\left[\sigma ({{{{{{{{\bf{u}}}}}}}}}^{m})/\sigma ({{{{{{{{\bf{u}}}}}}}}}_{t}^{m})\right]}^{2}$. Note that, to facilitate the PDE solution approximation highlighting the measurement data, we generally reduce the value of *α* in the pre-training stage by several times (e.g., 2–10) to relax the physics constraint. In ADO and post-training, the value of *α* is increased (e.g., *α* ~ *r*_*σ*_) to enhance the discovery of the PDE structure and fine tuning of PDE coefficients. However, we also find exception such as the *λ*-*ω* equations, where *α* in both pre-training and ADO stages should be set greater than the scaled ratio. It is likely due to the high resemblance between **u** and **v** in the spiral pattern, which can be alleviated if datasets from diverse IB/Cs are included in the measurements. Nevertheless, we have to mention that how to select this hyper-parameter is a common and critical open question in the PINN community.
- ${\beta }$: This hyper-parameter is the coefficient of the *ℓ*_0_ regularizer on **Λ** for the physics regression used in STRidge, which helps adaptively adjust the threshold tolerance in Supplementary Algorithm 1. We propose a Pareto front analysis strategy to estimate the value of *β* in order to best balance the physics loss and the equation sparsity. We first construct the sparse regression problem (see Eq. (6a)) solved by STRidge, where $\dot{{{{{{{{\bf{U}}}}}}}}}$ and **Φ** are evaluated based on the pre-trained DNN (with the trained network parameters denoted by ***θ***_0_). A grid search for *β* is then performed to obtain the graphical representation of the Pareto set (e.g., ${{{{{{{{\mathcal{L}}}}}}}}}_{p}({{{{{{{{\boldsymbol{\theta }}}}}}}}}_{0},{{{{{{{\mathbf{\Lambda }}}}}}}};{{{{{{{{\mathcal{D}}}}}}}}}_{c})$ vs. ∥**Λ**∥_0_). The optimal range of *β* can then be determined (see Supplementary Note 3.8). To avoid scaling issues, we further define $\beta =\kappa {{{{{{{{\mathcal{L}}}}}}}}}_{p}({{{{{{{{\boldsymbol{\theta }}}}}}}}}_{0},{{{{{{{{\mathbf{\Lambda }}}}}}}}}_{0};{{{{{{{{\mathcal{D}}}}}}}}}_{c})$, where *κ* is an auxiliary scaling variable determined by the Pareto front analysis and **Λ**_0_ denotes the pre-trained PDE coefficients.
- ${\gamma }$: This hyper-parameter used in pre-training (i.e., coefficient of the *ℓ*_1_ regularizer) is set to be a small value, e.g., 1 × 10^−7^. Our parametric study showed that this parameter is less important, which can also be set as zero although a small *γ* helps weakly promote the coefficient sparsity for PDE candidate terms.
- ${{n}_{{{\mbox{max}}}}}$: Based on our extensive tests, it is observed that the correct PDE structure can always be found within the first couple of ADO iterations. Hence, a safe value of 5–10 for the maximum number of ADO iterations (*n*_max_) will be sufficient to ensure convergence, e.g., we set *n*_max_ as 6 in all examples.

Other hyper-parameters (e.g., number of epochs, number of STRidge iterations, and the threshold increment in STRidge) used to activate Supplementary Algorithm 1 and Algorithm 2 are further discussed in detail in Supplementary Note 1.1 and 3.8.

### Initialization of trainable variables

Initiation of the heterogeneous trainable variables remains different. Specifically, the DNN weights are initialized based on Xavier Initialization, while the sparse PDE coefficients are uninformatively initialized either as zero or by uniformly sampling in [−1, 1].

### Selection of candidate functions

The library of candidate functions is a significant component in PINN-SR. Designing a a priori inclusive but not unnecessarily large library is a difficult task. On one hand, we prefer to make the candidate library as diverse as possible. On the other hand, balancing the increasing theoretical and computational complexity is crucial for applications. We believe that a specialized library hinged by our domain-specific knowledge and statistical experience can constrain the search space and reduce the complexity of PDE discovery. Although the higher the dimension of the library is, the more likely the exact terms will be uncovered from data. Nevertheless, a highly large-scale library (e.g., the number of components on the order of magnitude of ≥10^3^), essentially approximated by the DNN, is very likely to be rank deficient and have poor conditioning, in addition to the growing theoretical complexity and computational burden. Balancing these concerns and finding mathematical principles based on domain-specific knowledge to establish an efficient candidate library remain an open problem. Moreover, failing to include essential candidate functions will lead to false-positive discovery of parsimonious closed form of PDEs, despite that a “best-of-fit” form can be found (see Supplementary Note 3.2). Since the majority of well-known first-order PDEs with respect to time can be represented by linear combination of several active linear/nonlinear terms, we try to include as many as possible commonly seen terms following polynomial basis in this study.

## Supplementary information

Supplementary Information

## Data Availability

All the used datasets in this study are available on GitHub at https://github.com/isds-neu/EQDiscovery.

## Code availability

All the source codes to reproduce the results in this study are available on GitHub at https://github.com/isds-neu/EQDiscovery.
