# Supplementary material for: Physics-informed learning of governing equations from scarce data
Source: Nat Commun. 2021 Oct 21;12:6136. doi: 10.1038/s41467-021-26434-1 (PMC8531004; doi:10.1038/s41467-021-26434-1)
Supplement: Supplementary file 1 — Supplementary Information [file 41467_2021_26434_MOESM1_ESM.pdf]

Supplementary Information for:
Physics-informed learning of governing equations from scarce data

Zhao Chen[1], Yang Liu[2], and Hao Sun[3,4,1,5]

[1]Department of Civil and Environmental Engineering, Northeastern University, Boston, MA 02115, USA
[2]Department of Mechanical and Industrial Engineering, Northeastern University, Boston, MA 02115, USA
[3]Gaoling School of Artificial Intelligence, Renmin University of China, Beijing, 100872, China
[4]Beijing Key Laboratory of Big Data Management and Analysis Methods, Beijing, 100872, China
[5]Department of Civil and Environmental Engineering, MIT, Cambridge, MA 02139, USA

Contents

1 Alternating Direction Optimization (ADO): Algorithm **1**
1.1 Selection of hyper-parameters . . . 3

2 Examples **3**
2.1 Discovery of benchmark PDEs with single dataset . . . 4
2.1.1 Burgers' equation . . . 4
2.1.2 Kuramoto-Sivashinsky equation . . . 4
2.1.3 Nonlinear Schrödinger equation . . . 6
2.1.4 Navier-Stokes equation . . . 7
2.1.5 λ-ω type Reaction-Diffusion equations . . . 8
2.2 Comparison with SINDy . . . 11
2.3 Discovery of PDEs with multiple independent datasets . . . 12
2.3.1 Burgers' equation with shock behavior . . . 12
2.3.2 Fitzhugh-Nagumo type of Reaction-Diffusion equations . . . 13
2.4 Experimental discovery of cell migration and proliferation . . . 16

3 Discussion **17**
3.1 Simultaneous identification of unknown source . . . 18
3.2 Effect of missing candidate terms . . . 19
3.3 Noisy measurements and collocation points . . . 20
3.4 On the effect of non-Gaussian noise . . . 22
3.5 Optimal sensor placement . . . 23
3.6 Convergence history . . . 23
3.7 A parametric study on network size . . . 24
3.8 List of hyper-parameters used in the examples . . . 25
3.9 Other limitations . . . 25

This supplementary document provides a detailed description of the proposed algorithm, examples, and discussion of technical challenges for discovering closed-form partial differential equations (PDEs) from scarce and noisy data.

1 Alternating Direction Optimization (ADO): Algorithm

The proposed ADO algorithm for training the network of PINN-SR with sparse regression is outlined in Algorithm 1, where the STRidge sub-function (a sequential thresholding regression process that serves as a proxy for ℓ_0 regularization [1, 2]) is given in Algorithm 2.

Algorithm 1 The proposed ADO for network training: $[\boldsymbol{\theta}_{\text{best}}, \boldsymbol{\Lambda}_{\text{best}}] = \texttt{ADO}\left(\mathcal{D}_u, \mathcal{D}_c, \alpha, \gamma, \Delta\delta, n_{\max}, n_{\text{str}}\right)$

Input: Measurement data $\mathcal{D}_u$, collocation points $\mathcal{D}_c = \{\mathbf{x}_i, t_i\}_{i=1,2,...,N_c}$, relative weighting of loss functions α and γ, threshold tolerance increment $\Delta\delta$ for STRidge, maximum number of ADO iterations $n_{\max}$, and maximum number of STRidge cycles n_{str}.
we take a 2D system in a 2D domain as an example: $\mathbf{u} = \{u, v\}$ and $\mathbf{x} = \{x, y\}$

Split measurement data $\mathcal{D}_u$ into training-validation sets ($n_{\text{tr}}/n_{\text{va}} = 80/20$): $\mathcal{D}_u^{\text{tr}} \in \mathbb{R}^{n_{\text{tr}} \times 2}$ and $\mathcal{D}_u^{\text{va}} \in \mathbb{R}^{n_{\text{va}} \times 2}$. # $N_m = n_{\text{tr}} + n_{\text{va}}$

Split collocation points $\mathcal{D}_c$ into training-validation sets ($m_{\text{tr}}/m_{\text{va}} = 80/20$): $\mathcal{D}_c^{\text{tr}} \in \mathbb{R}^{m_{\text{tr}} \times 3}$ and $\mathcal{D}_c^{\text{va}} \in \mathbb{R}^{m_{\text{va}} \times 3}$. # $N_c = m_{\text{tr}} + m_{\text{va}}$

Initialize the *Tensor Graph* for the entire network.

Pre-train the network via combined Adam and L-BFGS with $\{\mathcal{D}_u^{\text{tr}}, \mathcal{D}_c^{\text{tr}}\}$, and validate the trained model with $\{\mathcal{D}_u^{\text{va}}, \mathcal{D}_c^{\text{va}}\}$, namely,

$$\{\hat{\boldsymbol{\theta}}_0, \hat{\boldsymbol{\Lambda}}_0\} = \arg\min_{\{\boldsymbol{\theta}, \boldsymbol{\Lambda}\}} \left\{\mathcal{L}_d(\boldsymbol{\theta}; \mathcal{D}_u) + \alpha\mathcal{L}_p(\boldsymbol{\theta}, \boldsymbol{\Lambda}; \mathcal{D}_c) + \gamma\|\boldsymbol{\Lambda}\|_1\right\}. \quad \text{\# pre-train the network; } \hat{\boldsymbol{\Lambda}}_0 = \{\hat{\boldsymbol{\lambda}}_0^u, \hat{\boldsymbol{\lambda}}_0^v\}$$

for $k = 1, 2, ..., n_{\max}$ **do**

Assemble the system states over the collocation points $\mathcal{D}_c^{\text{tr}}$ and $\mathcal{D}_c^{\text{va}}$:

$$\dot{\mathbf{U}}_u^{\text{tr}} = \bigcup_{i=1}^{N_c^{\text{tr}}} u_t\left(\hat{\boldsymbol{\theta}}_{k-1}; \mathbf{x}_i^{\text{tr}}, t_i^{\text{tr}}\right) \quad \text{and} \quad \dot{\mathbf{U}}_u^{\text{va}} = \bigcup_{i=1}^{N_c^{\text{tr}}} u_t\left(\hat{\boldsymbol{\theta}}_{k-1}; \mathbf{x}_i^{\text{va}}, t_i^{\text{va}}\right)$$
$$\dot{\mathbf{U}}_v^{\text{tr}} = \bigcup_{i=1}^{N_c^{\text{va}}} v_t\left(\hat{\boldsymbol{\theta}}_{k-1}; \mathbf{x}_i^{\text{tr}}, t_i^{\text{tr}}\right) \quad \text{and} \quad \dot{\mathbf{U}}_v^{\text{va}} = \bigcup_{i=1}^{N_c^{\text{va}}} v_t\left(\hat{\boldsymbol{\theta}}_{k-1}; \mathbf{x}_i^{\text{va}}, t_i^{\text{va}}\right).$$

Assemble the candidate library matrices over the collocation points $\mathcal{D}_c$, $\mathcal{D}_c^{\text{tr}}$ and $\mathcal{D}_c^{\text{va}}$:

$$\tilde{\boldsymbol{\Phi}} = \bigcup_{i=1}^{N_c} \boldsymbol{\phi}\left(\hat{\boldsymbol{\theta}}_{k-1}; \mathbf{x}_i, t_i\right), \quad \tilde{\boldsymbol{\Phi}}^{\text{tr}} = \bigcup_{i=1}^{N_c^{\text{tr}}} \boldsymbol{\phi}\left(\hat{\boldsymbol{\theta}}_{k-1}; \mathbf{x}_i^{\text{tr}}, t_i^{\text{tr}}\right) \quad \text{and} \quad \tilde{\boldsymbol{\Phi}}^{\text{va}} = \bigcup_{i=1}^{N_c^{\text{va}}} \boldsymbol{\phi}\left(\hat{\boldsymbol{\theta}}_{k-1}; \mathbf{x}_i^{\text{va}}, t_i^{\text{va}}\right).$$

Normalize candidate library matrices $\tilde{\boldsymbol{\Phi}}, \tilde{\boldsymbol{\Phi}}^{\text{tr}}$ and $\tilde{\boldsymbol{\Phi}}^{\text{va}}$ column-wisely ($j = 1, ..., s$) to improve matrix condition:

$$\boldsymbol{\Phi}_{:,j} = \tilde{\boldsymbol{\Phi}}_{:,j} / \left\|\tilde{\boldsymbol{\Phi}}_{:,j}\right\|_2, \quad \boldsymbol{\Phi}_{:,j}^{\text{tr}} = \tilde{\boldsymbol{\Phi}}_{:,j}^{\text{tr}} / \left\|\tilde{\boldsymbol{\Phi}}_{:,j}^{\text{tr}}\right\|_2 \quad \text{and} \quad \boldsymbol{\Phi}_{:,j}^{\text{va}} = \tilde{\boldsymbol{\Phi}}_{:,j}^{\text{tr}} / \left\|\tilde{\boldsymbol{\Phi}}_{:,j}^{\text{tr}}\right\|_2.$$

Determine ℓ_0 regularization parameters $\beta^u = \kappa\mathcal{L}_p^u\left(\hat{\boldsymbol{\theta}}_0, \hat{\boldsymbol{\lambda}}_0^u; \mathcal{D}_c^{va}\right)$ and $\beta^v = \kappa\mathcal{L}_p^v\left(\hat{\boldsymbol{\theta}}_0, \hat{\boldsymbol{\lambda}}_0^v; \mathcal{D}_c^{va}\right)$. # κ can be determined via a Pareto front analysis, e.g., $\kappa = 1$.

Initialize the error indices:

$$\hat{\epsilon}^u = \mathcal{L}_p^u\left(\hat{\boldsymbol{\theta}}_{k-1}, \hat{\boldsymbol{\lambda}}_{k-1}^u; \mathcal{D}_c^{va}\right) + \beta^u\left\|\hat{\boldsymbol{\lambda}}_{k-1}^u\right\|_0 \text{ and } \hat{\epsilon}^v = \mathcal{L}_p^v\left(\hat{\boldsymbol{\theta}}_{k-1}, \hat{\boldsymbol{\lambda}}_{k-1}^v; \mathcal{D}_c^{va}\right) + \beta^v\left\|\hat{\boldsymbol{\lambda}}_{k-1}^v\right\|_0.$$

Set the initial threshold tolerance $\delta_1 = \Delta\delta$.

for $iter = 1, 2, ..., n_{\text{str}}$ **do**

Run STRidge as shown in Algorithm 2 to determine:

$$\tilde{\boldsymbol{\lambda}}^u = \texttt{STRidge}\left(\dot{\mathbf{U}}_u^{\text{tr}}, \boldsymbol{\Phi}^{\text{tr}}, \delta_{iter}\right) \quad \text{and} \quad \tilde{\boldsymbol{\lambda}}^v = \texttt{STRidge}\left(\dot{\mathbf{U}}_v^{\text{tr}}, \boldsymbol{\Phi}^{\text{tr}}, \delta_{iter}\right).$$

Update the error indices:

$$\epsilon^u = \mathcal{L}_p^u\left(\hat{\boldsymbol{\theta}}_{k-1}, \tilde{\boldsymbol{\lambda}}^u; \mathcal{D}_c^{va}\right) + \beta^u\left\|\tilde{\boldsymbol{\lambda}}^u\right\|_0 \text{ and } \epsilon^v = \mathcal{L}_p^v\left(\hat{\boldsymbol{\theta}}_{k-1}, \tilde{\boldsymbol{\lambda}}^v; \mathcal{D}_c^{va}\right) + \beta^v\left\|\tilde{\boldsymbol{\lambda}}^v\right\|_0.$$

if $\epsilon^u \leqslant \hat{\epsilon}^u$ or $\epsilon^v \leqslant \hat{\epsilon}^v$ (run in parallel) **then**

Increase threshold tolerance with increment: $\delta_{iter+1} = \delta_{iter} + \Delta\delta$.

else

Decrease threshold tolerance increment $\Delta\delta = \Delta\delta/1.618$.

Update threshold tolerance with the new increment $\delta_{iter+1} = \max\{\delta_{iter} - 2\Delta\delta, 0\} + \Delta\delta$.

end if

end for

Return and re-scale the current best solution from STRidge cycles: $\hat{\boldsymbol{\Lambda}}_k = \left\{\tilde{\boldsymbol{\lambda}}^u, \tilde{\boldsymbol{\lambda}}^v\right\}$. # re-scaling due to normalization of $\boldsymbol{\Phi}$

Train the DNN via combined Adam and L-BFGS with $\{\mathcal{D}_u^{\text{tr}}, \mathcal{D}_c^{\text{tr}}\}$, and validate the trained model with $\{\mathcal{D}_u^{\text{va}}, \mathcal{D}_c^{\text{va}}\}$, namely,

$$\hat{\boldsymbol{\theta}}_k = \arg\min_{\boldsymbol{\theta}} \left\{\mathcal{L}_d(\boldsymbol{\theta}; \mathcal{D}_u) + \alpha\mathcal{L}_p\left(\boldsymbol{\theta}, \hat{\boldsymbol{\Lambda}}_k; \mathcal{D}_c\right)\right\}. \quad \text{\# train DNN given } \hat{\boldsymbol{\Lambda}}_k \text{ as known}$$

end for

Output: the best solution $\boldsymbol{\theta}_{\text{best}} = \hat{\boldsymbol{\theta}}_{n_{\max}}$ and $\boldsymbol{\Lambda}_{\text{best}} = \hat{\boldsymbol{\Lambda}}_{n_{\max}}$

Algorithm 2 Sequential threshold ridge regression (STRidge): $\hat{\boldsymbol{\lambda}} = \texttt{STRidge}(\dot{\mathbf{U}}, \mathbf{\Phi}, \delta)$

1: **Input:** Time derivative vector $\dot{\mathbf{U}}$, candidate function library matrix $\mathbf{\Phi}$, and threshold tolerance δ.
2: Inherit coefficients $\hat{\boldsymbol{\lambda}}$ from the DNN pre-training or the previous update.
3: **repeat**
4: Determine indices of coefficients in $\hat{\boldsymbol{\lambda}}$ falling below or above the sparsity threshold δ:

$$\mathcal{I} = \{i \in \mathcal{I} : |\hat{\lambda}_i| < \delta\} \text{ and } \mathcal{J} = \{j \in \mathcal{J} : |\hat{\lambda}_j| \geqslant \delta\}.$$

5: Enforce sparsity to small values by setting them to zero: $\hat{\boldsymbol{\lambda}}_{\mathcal{I}} = \mathbf{0}$.
6: Update remaining non-zero values with ridge regression:

$$\hat{\boldsymbol{\lambda}}_{\mathcal{J}} = \arg\min_{\boldsymbol{\lambda}_{\mathcal{J}}} \{\|\mathbf{\Phi}_{\mathcal{J}}\boldsymbol{\lambda}_{\mathcal{J}} - \dot{\mathbf{U}}\|_2^2 + 1 \times 10^{-5}\|\boldsymbol{\lambda}_{\mathcal{J}}\|_2^2\}. \quad \text{\# the parameter } 1 \times 10^{-5} \text{ is small and tunable}$$

7: **until** maximum number of iterations reached.
8: **Output:** The best solution $\hat{\boldsymbol{\lambda}} = \hat{\boldsymbol{\lambda}}_{\mathcal{I}} \cup \hat{\boldsymbol{\lambda}}_{\mathcal{J}}$

1.1 Selection of hyper-parameters

The criteria for selecting hyper-parameters $\{\alpha, \beta, \gamma, n_{\text{max}}\}$ have been introduced in Main Text (see Method). Other required hyper-parameters are selected based on the criteria below.

- Maximum Number of Epochs: Our intuition of selecting the number of epochs for DNN training follows the general practice: the network should be sufficiently trained. It depends on the specific problem complexity and the number of trainable parameters. The pre-training typically consists of up to $10^4 \sim 10^5$ epochs for Adam and/or BFGS, while, in each ADO iteration, 10^3 epochs for Adam are used. Post-training might consist of up to 3×10^4 epochs for Adam and/or BFGS.
- Maximum Number of STRidge Cycles (n_{str}): We set the number of STRidge cycles large enough (e.g., 100) to allow the algorithm to heuristically locate an optimal threshold.
- Threshold Tolerance Increment ($\Delta\delta$): The role of $\Delta\delta$ is to adaptively determine δ that introduces proper sparsity in the discovered equation. Since the optimal threshold is automatically determined and normalized in STRidge, we take a small positive value (e.g., $\Delta\delta = 1$) as long as the number of STRidge cycles is sufficient. However, in the case of complex-value systems (such as the nonlinear Schrödinger equation), since the threshold is compared with the modulus of complex numbers, we find empirically setting $\Delta\delta$ as 100 is more proper.

The list of hyper-parameters used in all examples in the paper is presented in Section 3.8.

2 Examples

We observe the efficacy and robustness of our methodology on a group of canonical PDEs used to represent a wide range of physical systems with nonlinear, periodic and/or chaotic behaviors. In particular, we discover the closed forms of Burgers', Kuramoto-Sivashinsky (KS), nonlinear Schrödinger, Navier-Stokes (NS), and λ-ω Reaction-Diffusion (RD) equations from scarce and noisy time-series measurements recorded by a number of sensors at fixed locations from a single I/BC. Gaussian white noise is added to the synthetic response with the noise level defined as the root-mean-square ratio between the noise and the exact solution. To demonstrate the "root-branch" network for discovery of PDE(s) based on multiple independent datasets sampled under different I/BCs, we consider (1) the 1D Burgers' equation with light viscosity that exhibits a shock behavior, and (2) a 2D Fitzhugh-Nagumo (FN) type reaction-diffusion system that describes activator-inhibitor neuron activities excited by external stimulus. At last, we test our framework on the experimental

data of cell migration and proliferation. Our method is also compared with SINDy (the PDE-FIND approach presented in [2]) which is also presented herein. The identification error is defined as the average relative error of the identified non-zero PDE coefficients with respect to the ground truth, which is used to evaluate the accuracy of the discovered PDEs for the following examples. Simulations in this paper are performed on a workstation with 28 Intel Core i9-7940X CPUs and 2 NVIDIA GTX 1080Ti GPU cards (otherwise, noted separately).

2.1 Discovery of benchmark PDEs with single dataset

2.1.1 Burgers' equation

We first consider a dissipative system with the dynamics governed by a 1D viscous Burgers' equation expressed as

$$u_t = -uu_x + \nu u_{xx}$$

where u is a field variable, x and t are the spatial and temporal coordinates, and ν denotes the diffusion coefficient. The equation describes the decaying stationary viscous shock of a system after a finite period of time, commonly found in simplified fluid mechanics, nonlinear acoustics, gas dynamics and traffic flow. In this work, solution for the Burgers' Equation is from an open dataset [2], in which the diffusion coefficient ν is assumed to be 0.1 and u is discretized into 256 spatial grid points for 101 time steps with a Gaussian initial condition. In particular, 10 sensors are randomly placed at fixed locations among the 256 spatial grid points to record the wave response for 101 time steps, leading to 3.19% of the dataset used in [2]. Among all the measurements, 80% are allocated for training purpose and the rest 20% for validation. A total number of 5×10^4 collocation points, e.g., in the pair of $\{x, t\}$, are sampled by the Latin hypercube sampling [3]. A group of 16 candidate functions ($\boldsymbol{\phi} \in \mathbb{R}^{1\times16}$) are used to reconstruct the PDE, consisting of polynomial terms (u, u^2, u^3), derivatives (u_x, u_{xx}, u_{xxx}) and their multiplications whose coefficient vector $\mathbf{\Lambda}$ is initialized uninformatively as zeros. The fully connected DNN has 8 hidden layers and a width of 20 neuron nodes in each layer where activation functions are hyperbolic tangent, weights are initialized based on Glorot normal distribution [4] and biases are initialized as zeros. The training efforts are performed via up to 1×10^4 epochs of L-BFGS for pre-training and 6 ADO iterations. In each ADO iteration, we use 1000 epochs of Adam to train the DNN for alternation with STRidge. The discovered equation for the dataset with 10 % noise reads:

$$u_t = -1.009uu_x + 0.099u_{xx}$$

where the aggregated relative identification error for all non-zero elements in $\mathbf{\Lambda}$ is $0.88 \pm 0.03\%$. Despite that only 3.19% subsampled responses are measured, the PINN-SR approach can accurately extrapolate the full-field solution with a ℓ_2 error of 1.32% (see Fig. 1A). Fig. 1B shows the comparison of spatial and temporal snapshots between the predicted and the exact solutions which agree extremely well.

2.1.2 Kuramoto-Sivashinsky equation

Another dissipative system with intrinsic instabilities is considered, governed by the 1D Kuramoto-Sivashinsky (KS) equation:

$$u_t = -uu_x - u_{xx} - u_{xxxx}$$

where the reverse diffusion term $-u_{xx}$ leads to the blowup behavior while the fourth-order derivative u_{xxxx} introduces chaotic patterns as shown in Fig. 2B, making an ideal test problem for equation

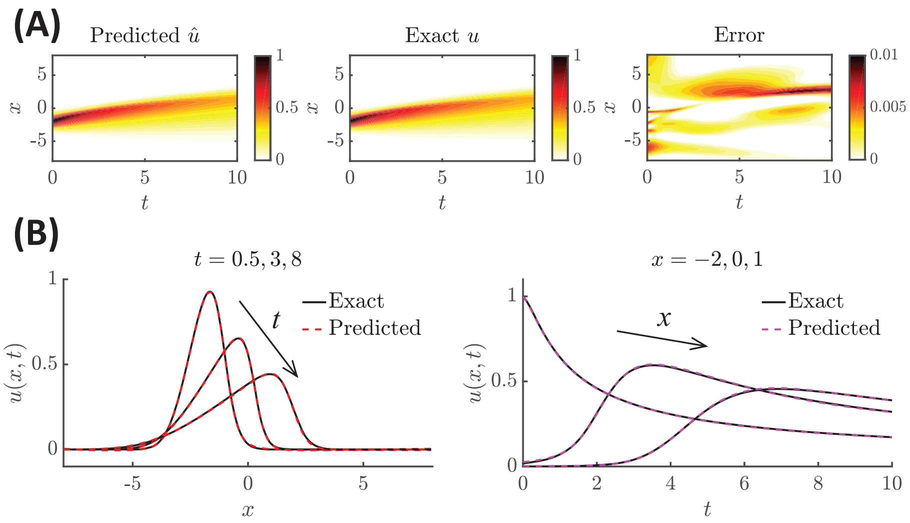

Figure 1: Discovery of Burgers' equation for data with 10% noise: (A) The predicted response in comparison with the exact solution with the prediction error. (B) Comparison of spatial and temporal snapshots between the predicted and the exact solutions. The relative full-field ℓ_2 error of the prediction is 1.32%.

discovery. Starting with a smooth initial condition, the KS system evolves to an unstable laminar status due to the highly nonlinear terms including the high-order derivative. The KS equation is widely used to model the instabilities in laminar flame fronts and dissipative trapped-ion modes among others. We subsample the open dataset [2] by randomly choosing 320 points from the 1024 spatial grid nodes as fixed sensors and record the wave response for 101 time steps, occupying about 12.6% of the original dataset. The training/validation measurements are separated based on an 80-20 principle. A set of 2×10^4 collocation points, generated using the Latin hypercube sampling in the spatiotemporal domain, are employed to evaluate the residual physics loss. A library of 36 candidate functions are used to construct the PDE, consisting of polynomials (u, u^2, u^3, u^4, u^5), derivatives $(u_x, u_{xx}, u_{xxx}, u_{xxxx}, u_{xxxxx})$ and their multiplications, whose initial coefficients are uninformatively chosen to be zeros. The DNN architecture has 8 hidden layers each with 40 nodes whose activation functions and initialization are the same as the previous Burgers' case. The training efforts are performed via 8×10^4 epochs of Adam and up to 8×10^4 epochs of L-BFGS for pre-training and 6 ADO iterations. In each ADO iteration, we use 1000 epochs of Adam to train the DNN for alternation with STRidge. As for post-training, 2×10^4 Adam epochs and up to 2×10^4 L-BFGS epochs are used.

It is notable that the chaotic behavior poses significant challenges in approximating the full-field spatiotemporal derivatives, especially the high-order u_{xxxx}, from poorly measured data for discovery of such a PDE. Existing methods (for example the family of SINDy methods [2, 5]) eventually fail in this case given very coarse and noisy measurements. Nevertheless, the PINN-SR approach successfully distils the closed form of the KS equation from subsampled sparse data even with 10% noise:

$$u_t = -0.991uu_x - 0.990u_{xx} - 0.990u_{xxxx}$$

where the coefficients have an average relative error, for all non-zero elements in $\mathbf{\Lambda}$, of $0.94 \pm 0.05\%$. Although the available measurement data are sparsely sampled in the spatiotemporal domain under a high-level noise corruption, the predicted full-field wave by the trained PINN-SR also agrees well with the exact solution with a relative ℓ_2 error of 2.14% (Fig. 2A). The spatial and temporal snapshots of the predicted response match seamlessly the ground truth as shown in Fig. 2B.

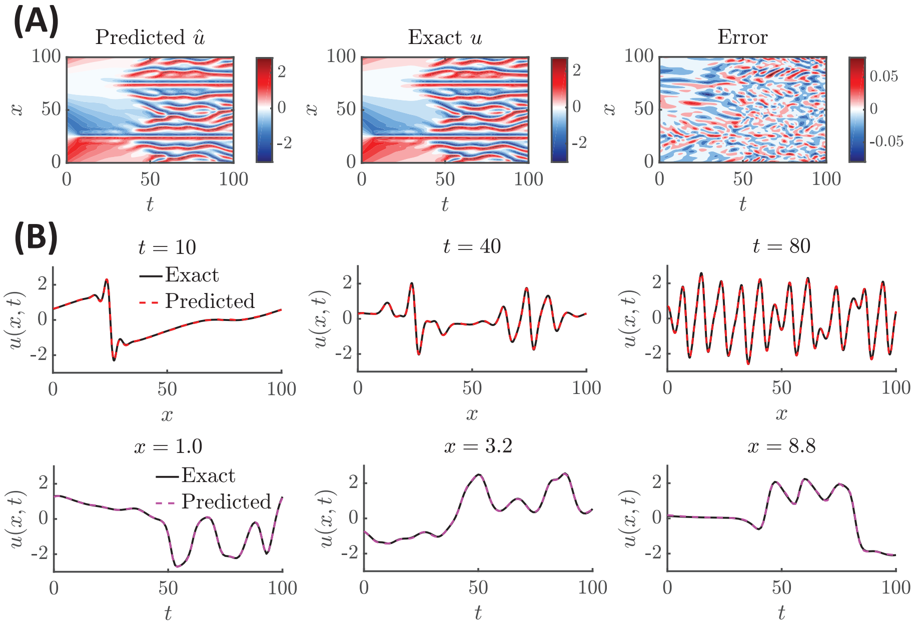

Figure 2: Discovery of the KS equation for data with 10% noise: (A) The predicted response compared with the exact solution. (B) Comparison of spatial and temporal snapshots between the predicted and the exact solutions. The relative full-field ℓ_2 error of the prediction is 2.14%.

2.1.3 Nonlinear Schrödinger equation

In the third example, we discover the nonlinear Schrödinger equation, originated as a classical wave equation, given by

$$iu_t = -0.5u_{xx} - |u|^2 u$$

where u is a complex field variable. This well-known equation is widely used in modeling the propagation of light in nonlinear optical fibers, Bose-Einstein condensates, Langmuir waves in hot plasmas, and so on. The solution to this Schrödinger equation is simulated based on a Gaussian initial condition with the problem domain meshed into 512 spatial points and 501 temporal steps, while the measurements are taken from 256 randomly chosen spatial "sensors" for 375 time instants, resulting in 37.5% data used in [2] for uncovering the closed form of the equation. A library of 40 candidate functions are used for constructing the PDE, varying among polynomial functions (u, u^2, u^3), absolute values $(|u|, |u|^2, |u|^3)$, derivatives (u_x, u_{xx}, u_{xxx}) and their combination, whose coefficients are initialized as zeros. Since the function is complex valued, we model separately the real part (u_R) and the imaginary part (u_I) of the solution in the output of the DNN, assemble them to obtain the complex solution $u = u_R + iu_I$, and construct the complex valued candidate functions for discovery. To avoid complex gradients in optimization, we compute the mean square error of the modulus $|u|$ for the residual physics loss $\mathcal{L}_p$. The fully connected DNN has 8 hidden layers and a width of 40 neuron nodes in each layer with the same activation functions and initialization for network's weights and biases as previous. The pre-training takes 8×10^4 epochs of Adam (with at most 1.6×10^5 epochs of L-BFGS) followed by 6 ADO iterations. In each ADO iteration, we use 1×10^3 Adam epochs to train the DNN for alternation with STRidge. Afterwards, we further conduct a post-training of 10^4 epochs of Adam and up-to 10^4 epochs of L-BFGS to tune the non-zero PDE coefficients.

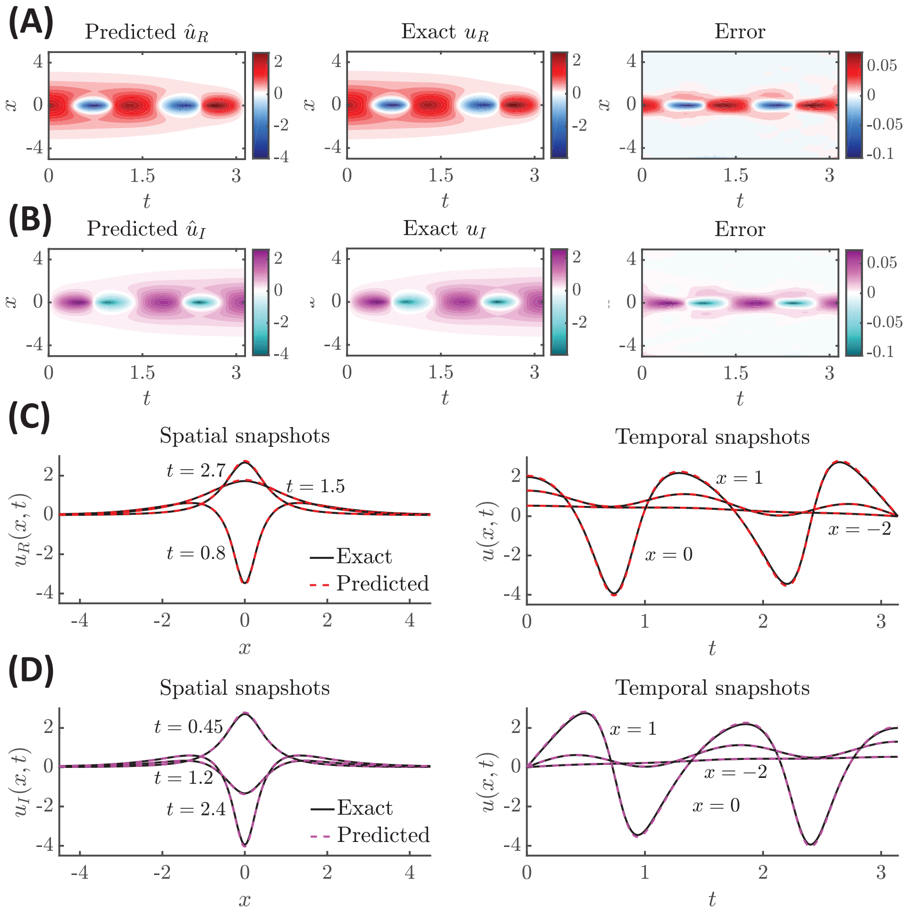

Figure 3: Discovery of nonlinear Schrödinger equation for a dataset with 10% noise: (A) and B) The predicted real-part (A) and imaginary-part (B) responses compared with the exact solution. (C and D) Comparison of spatial and temporal snapshots between the predicted and the exact solutions for the real part (C) and imaginary part (D). The average relative full-field ℓ_2 error of the real and imaginary prediction is 0.26%.

The discovered equation under 10 % noise is written as

$$iu_t = -0.501u_{xx} - 1.000|u|^2u$$

where the average relative error for non-zero coefficients is $0.08 \pm 0.03\%$. The predicted full-field response, for both real and imaginary parts, matches well the exact solution with a slight relative ℓ_2 error of about 0.26% (Fig. 3A and B). The comparison of spatiotemporal snapshots between the predicted and the exact solutions for the real part (Fig. 3C) and imaginary part (Fig. 3D) also shows almost perfect agreement.

2.1.4 Navier-Stokes equation

We consider a 2D fluid flow passing a circular cylinder with the local rotation dynamics (see Fig. 4). For incompressible and isotropic fluids which also have conservative body forces, the well-known Navier-Stokes vorticity equation reads

$$w_t = -uw_x - vw_y + 0.01w_{xx} + 0.01w_{yy}$$

where w is the spatiotemporally variant vorticity, $\mathbf{u} = \{u, v\}$ denotes the fluid velocities at Reynolds number 100. The full-field solution to the NS vorticity equation is obtained using the immersed

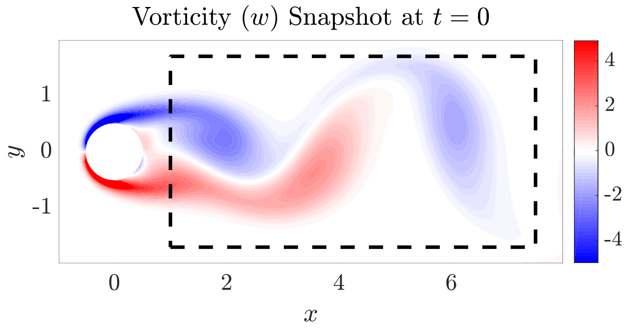

Figure 4: Vorticity field $w(\mathbf{x}, t)$ at $t = 0$ for a steady flow passing a cylinder. Measurements are sampled from the the boxed area surrounded by the dashed line.

boundary projection method [6]. The dimensionless domain is discretized into a 499×199 spatial grid and 151 time steps. The cylinder has a unit diameter and the input flow from the left side has a unit velocity. Measurements of velocities $\{u, v\}$ and vorticity w are taken at 500 random spatial locations lasting 60 time steps in the boxed area behind the cylinder as shown in Fig. 4, namely 0.20% subsamples from the total dataset and 1/10 of the data used in [2]. The residual physics loss is evaluated on 6×10^4 collocation points randomly sampled in the spatiotemporal doamin using the Latin hypercube sampling method. The library of candidate functions consists of 60 components including polynomial terms $(u, v, w, uv, uw, vw, u^2, v^2, w^2)$, derivatives $(w_x, w_y, w_{xx}, w_{xy}, w_{yy})$ and their combination, whose coefficients are initialized as zeros. The latent output in the DNN contains u, v and w. The DNN has 8 fully connected hidden layers and a width of 60 nodes in each layer with the same activation functions and initialization methods as previous. The pre-training takes 5×10^3 epochs of Adam (with additional L-BFGS tuning up to 1×10^4 epochs) followed by 6 ADO iterations. In each ADO iteration, we use the Adam optimizer with 1000 epochs to train the DNN for each alternation within STRidge.

The discovered NS vorticity equation for the case of 10% noise is given as follows

$$w_t = -0.996uw_x - 0.991vw_y + 0.010w_{xx} + 0.010w_{yy}$$

where the aggregated relative identification error for all non-zero elements in $\mathbf{\Lambda}$ is $1.22 \pm 0.69\%$. It is encouraging that the uncovered vorticity equation is almost identical to the ground truth, for both the derivative terms and their coefficients, even under 10% noise corruption. The vorticity patterns and magnitudes are also well predicted as indicated by multiple spatial snapshots at different time instants ($t = 0.2, 7.6, 15, 22.4, 29.8$) shown in Fig. 5$A$ in comparison with the exact solution (Fig. 5B, with small errors as depicted in Fig. 5C). Note that the response in these snapshots is not used in training the network. The ℓ_2 error of the predicted full-field vorticity response is about 2.58%. This example provides a compelling test case for the proposed PINN-SR approach which is capable of discovering the closed-form NS equation with scarce and noisy data.

2.1.5 λ-ω type Reaction-Diffusion equations

The examples discussed previously are low-dimensional (1D) models with limited complexity. We herein consider a λ-ω reaction-diffusion (RD) system in a 2D domain with the pattern forming behavior governed by two coupled PDEs:

$$\begin{cases} u_t = 0.1u_{xx} + 0.1u_{yy} - uv^2 - u^3 + v^3 + u^2v + u \\ v_t = 0.1v_{xx} + 0.1v_{yy} - uv^2 - u^3 - v^3 - u^2v + v \end{cases}$$

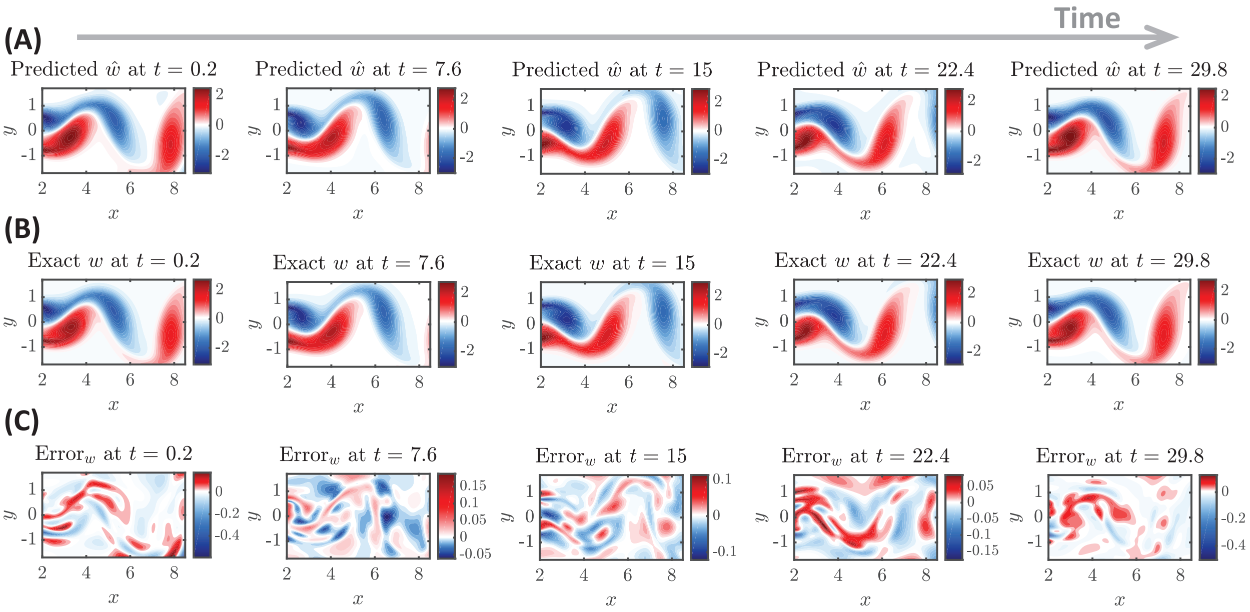

Figure 5: Discovery of NS equation for data with 10% noise: $(A\text{-}C)$ Vorticity snapshots at different time instants $(t = 0.2, 7.6, 15, 22.4, 29.8)$ for the prediction (A), the exact solution (B) and the prediction error (C). Note that response at these time instants are not included in dataset for training the PINN-SR model and equation discovery. The relative full-field ℓ_2 error of the prediction $\hat{w}$ is about 2.58%.

where u and v are two field variables. The λ-ω equations are typically used to describe the multiscale phenomenon of local reactive transformation and the global diffusion in chemical reactions, with wide applications in pattern formation [7], biological morphogenesis [8], and ecological invasions [9], among others. The λ-ω equations exhibit a wide range of behaviors including wave-like phenomena and self-organized patterns found in chemical and biological systems. The binomial system is also called an activator-inhibitor system because one state variable encourages the increase of both states while the other state component inhibits their growth. The particular λ-ω equations in this test example display spiral waves subjected to periodic boundary conditions. The domain for generating the solution is divided into 65,536 (256×256) spatial points with 201 time steps. We take randomly 2,500 spatial points as fixed sensors recording the wave response for 15 randomly sampled time steps, leading to 1/4 of the subsampled dataset used in [2] and 0.29% of the total data. We sample 8×10^4 collocation points using the Latin hypercube sampling to evaluate the residual physics loss. A total of 110 candidate functions are employed, including polynomials up to the 3rd order $(u, v, u^2, v^2, uv, u^3, u^2v, uv^2, v^3)$, derivatives up to the 2nd order $(u_x, u_y, v_x, v_y, u_{xx}, u_{xy}, u_{yy}, v_{xx}, v_{xy}, v_{yy})$ and their combination, for the sparse discovery of the two PDEs, whose coefficients are initialized as zeros. Since the system dimension is relatively high, we enhance the discovery by post-training (post-tuning) of the DNN and the uncovered non-zero PDE coefficients, after the ADO stage, resulting in refined/improved discovery. The DNN has 8 fully connected hidden layers and a width of 60 nodes in each layer, whose activation functions and initializations are the same as previous case studies. Due to the high dimensionality of the system and a large candidate library, we run this case on a workstation with an NVIDIA Tesla V100 GPU card (32 GB). The pre-training takes 1×10^4 epochs of Adam (with additional L-BFGS tuning up to 4×10^4 epochs) followed by 6 ADO iterations. In each ADO iteration, we use 1×10^3 Adam epochs to train the DNN for each alternation within STRidge. After discovering the PDE equation structure, we further use 1×10^4 Adam and up to 1×10^4 L-BFGS to refine DNN and equation

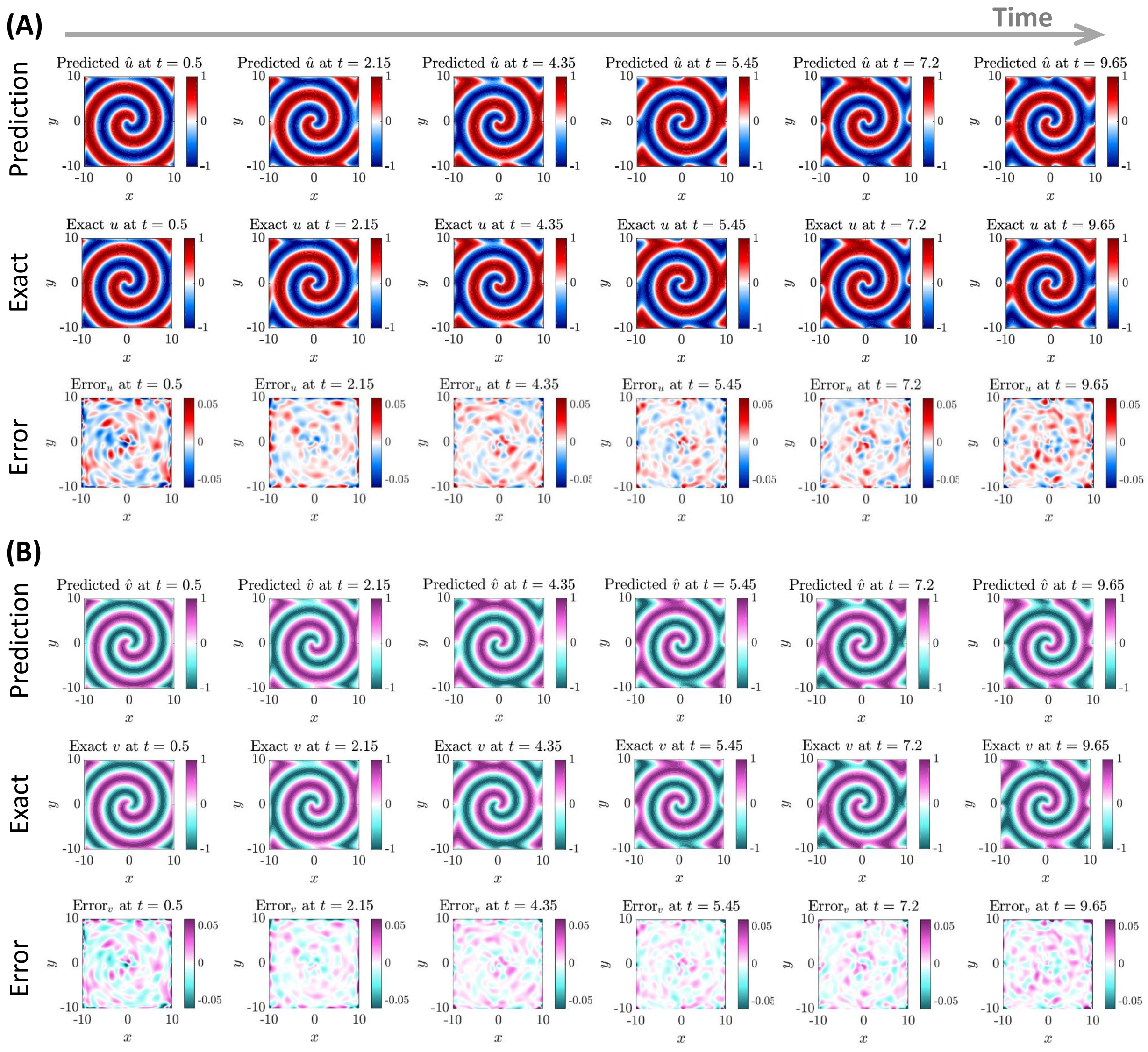

Figure 6: Discovery of λ-ω equations for a dataset with 10% noise: (A and B) The response snapshots u (A) and v (B) at different time instants ($t = 0.5, 2.15, 4.35, 5.45, 7.2, 9.65$), showing the predictions and the exact solutions, as well as the prediction error maps. The average relative full-field ℓ_2 error of the predicted $\hat{u}$ and $\hat{v}$ is about 2.14%.

coefficients.

The reconstructed equations for the case of 10% noise are given by

$$\begin{cases} u_t = 0.097u_{xx} + 0.097u_{yy} - 0.955uv^2 - 0.964u^3 + 0.997v^3 + 0.997u^2v + 0.964u \\ v_t = 0.099v_{xx} + 0.101v_{yy} - 1.009uv^2 - 1.002u^3 - 0.982v^3 - 0.989u^2v + 0.982v \end{cases}$$

where the the average relative error for all non-zero coefficients is $1.84 \pm 1.48\%$. The predicted response snapshots by the trained PINN-SR at different time instants, e.g., $t = \{0.5, 2.15, 4.35, 5.45, 7.2, 9.65\}$, are shown in in Fig. 6$A$ and B, which are very close to the ground truth (the errors are distributed within a small range). This example shows especially the great ability and robustness of our method for discovering governing PDEs for high-dimensional systems from highly noisy data. The average relative full-field ℓ_2 error of the predicted $\hat{u}$ and $\hat{v}$ is 2.14%.

2.2 Comparison with SINDy

We have performed the comparison study between the proposed PINN-SR approach and the state-of-the-art PDE-FIND method (an extended version of SINDy) [2], in the context of different levels of data size and noise. We test the five PDEs described previously and summarize the discovery errors for the sparse coefficients in Table 1. The error is defined as the average relative error of the identified non-zero PDE coefficients with respect to the ground truth. If the terms in the PDEs are discovered incorrectly, we mark it as "Fail". It is seen from Table 1 that the proposed PINN-SR approach is capable of correctly uncovering the closed-form PDEs for all cases, regardless of the varying levels of data size and noise, which demonstrates excellent robustness. Although PDE-FIND shows great success in PDE discovery with negligible error for large and clean (or approximately noise-free) measurement data, this method eventually fails when the level of data scarcity and/or noise increases. In general, PDE-FIND relies on the strict requirement of measurement quality and quantity. However, PINN-SR is able to alleviate and resolve this limitation thanks to the combination of the strengths of DNNs for rich representation learning of nonlinear functions, automatic differentiation for accurate derivative calculation as well as ℓ_0 sparse regression. In addition, the use of collocation points introduces additional "pseudo datasets", compensates indirectly the scarcity of measurement data, and enriches the constraint for constructing the closed form of PDEs.

We must admit that, since our method involves DNN training, the computational cost is much higher compared with SINDy (e.g., 553 seconds for ours vs. 2 seconds for SINDy, in the Burgers' example). However, the critical bottleneck of SINDy lies in its requirement of large high-quality (clean) structured measurement data, owing to its use of numerical differentiation, which poses

Table 1: Summary of the PINN-SR discovery results in comparison with PDE-FIND [2] for canonical models.

PDE name	Method	Error (noise 0%)	Error (noise 1%)	Error (noise 10%)	# of Measurement points
Burgers'	PINN-SR	0.01±0.01%	0.19±0.11%	0.88 ± 0.03%	∼1k
	PDE-FIND	Fail	Fail	Fail	∼1k
		0.15±0.06%	0.80±0.60%	Fail	∼26k
KS	PINN-SR	0.07±0.01%	0.61±0.04%	0.94 ± 0.05%	∼32k
	PDE-FIND	35.75±16.30%	Fail	Fail	∼32k
		1.30±1.30%	52.00±1.40%	Fail	∼257k
Schrödinger	PINN-SR	0.09±0.04%	0.65±0.29%	0.08±0.03%	∼96k
	PDE-FIND	Fail	Fail	Fail	∼96K
		0.05±0.01%	3.00±1.00%	Fail	∼257k
NS	PINN-SR	0.66±0.72%	0.86±0.63%	1.22±0.69%	∼30k
	PDE-FIND	Fail	Fail	Fail	∼30K
		1.00±0.20%	7.00±6.00%	Fail	∼300k
λ-ω RD	PINN-SR	0.07±0.08%	0.25±0.30%	1.84 ± 1.48%	∼37.5k
	PDE-FIND	Fail	Fail	Fail	∼37.5k
		0.02±0.02%	Fail	Fail	∼150k

Note: KS, NS and RD refer to the Kuramoto-Sivashinsky, Navier-Stokes and λ-ω Reaction-Diffusion PDEs. Gaussian white noise is added to the synthetic response with the noise level defined as the root-mean-square ratio between the noise and exact solution. "Fail" denotes failure in discovery of the sparse PDE coefficients. The identification error is defined as the average relative error of the identified non-zero PDE coefficients with respect to the ground truth.

critical limitation of SINDy in practical applications where data is sparse and noisy (e.g., the experimental data in the cell migration and proliferation example, discussed in Section 2.4). While PINN-SR is more computationally costly, it is capable of producing accurate discovery even in the presence of sparse and noisy datasets. There is obviously a trade-off between computational efficiency and need of high-quality data. Furthermore, this issue of longer computational time can be well managed through parallel computing on a powerful GPU platform and remains a less important concern compared to the aim for successful discovery of correct underlying PDEs.

2.3 Discovery of PDEs with multiple independent datasets

2.3.1 Burgers' equation with shock behavior

We consider to discover the previously discussed Burgers' equation (see Section 2.1.1) with a small diffusion/viscosity parameter, expressed as

$$u_t = -uu_x + \frac{0.01}{\pi}u_{xx}$$

based on datasets generated by imposing three different I/BCs. The small diffusion coefficient $0.01/\pi \approx 0.0032$ creates creates shock formation in a compact area with sharp gradient and poses notorious difficulty for many numerical methods to resolve, which could challenge the DNN's approximation ability and thus affect the discovery. The three I/BCs used for data generation include:

$$\begin{aligned}
&\text{I/BC 1: } u(x,0) = -\sin(\pi x), u(-1,t) = u(1,t) = 0\\
&\text{I/BC 2: } u(x,0) = \mathcal{G}(x), u(-1,t) = u(1,t) = 0\\
&\text{I/BC 3: } u(x,0) = -x^3, u(-1,t) = 1, u(1,t) = -1
\end{aligned}$$

where $\mathcal{G}$ denotes a Gaussian function. The ground truth solution is simulated by MATLAB function `pdede` in a spatiotemporal domain $\Omega \times [0,T] = [-1,1]_{d=200} \times [0,1]_{d=1000}$. For all I/BCs, we assume that there are 30 sensors randomly deployed in space measuring the wave traveling (e.g., u) for 500 time instants (7.5% of the total grid points). A denser sensor grid is needed herein, compared with the previous Burgers' example, in order to capture the shock behaviors. All measurements are polluted with 10% Gaussian noise. The noisy measurements are depicted in Fig. 7*A* for the three datasets. For visualization purpose, we only draw a handful of signals out of a total of 30 time series for each I/BC.

We design a "root-branch" DNN: the root takes the spatiotemporal coordinates $\{x,t\}$ as input followed by 4 hidden layers of 20 nodes, while each of the three branches is separately connected to the last hidden layer of the root followed by 4 hidden layers of 30 nodes before the output layer, whose activation functions and initializations are the same as the previous. The motivation for this design is that the branch nets can capture the solution difference due to different I/BCs while the shared root net learns the common response patterns that obey the unique Burgers' equation. Note that although we have three distinctive solution approximations, we stack them into one candidate library followed by a unified form of PDE. Therefore, we can combine the information from three datasets to discover one physics equation. A group of 4.5×10^4 collocation points are generated by the Latin hypercube sampling strategy [3] for determining the residual physics loss. The PDE library consists of 16 candidate functions, exactly the same as the Burgers' case in Section 2.1.1, whose coefficients are initialized as zeros. Due to the size of the composite deep neural networks, we run this case on a workstation with an NVIDIA Tesla V100 GPU card (32 GB). The training efforts include up to 8×10^4 epochs pretraining by L-BFGS followed by 6 ADO iterations. In each ADO

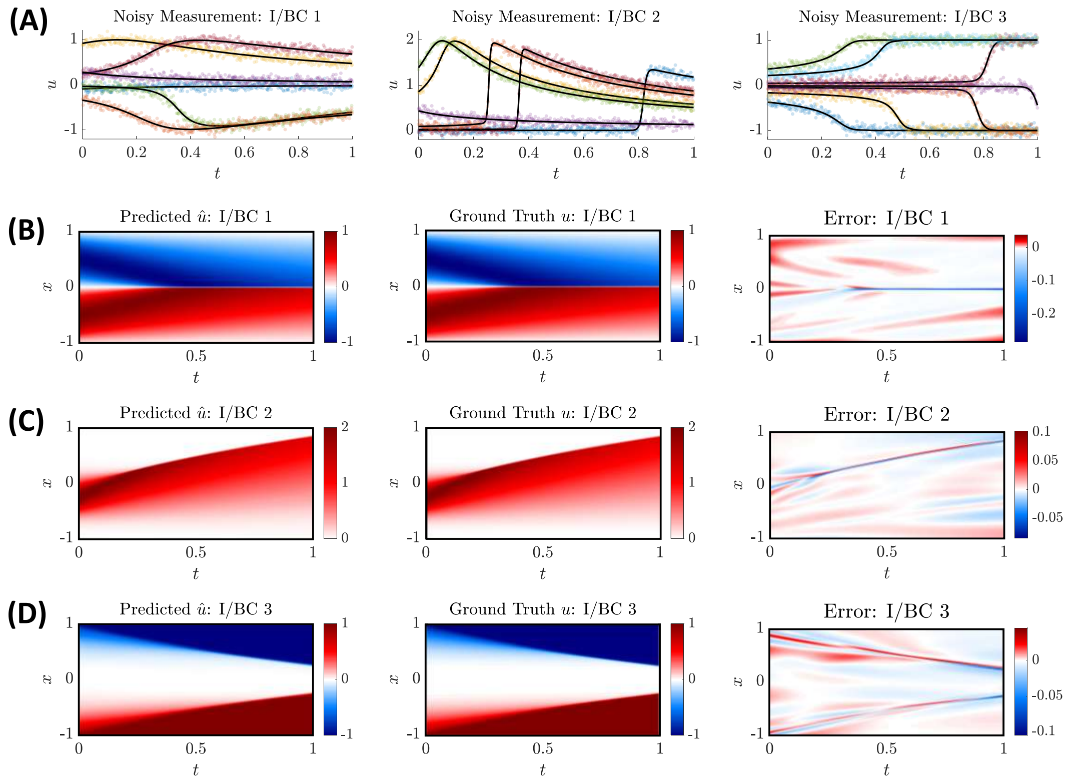

Figure 7: Discovery of Burgers' equation with small viscosity based on datasets sampled under three I/BCs with 10% noise: (*A*) Visualization of noisy measurements for the three datasets. Note that there are 30 sensors and only a few are illustrated in this figure. (*B-D*) The predicted response in comparison with the exact solution for three I/BCs. The relative full-field ℓ_2 error of all the stacked predictions is 0.65%.

iteration, we use 1×10^3 Adam epochs to train the DNN in synergy with STRidge. The discovered PDE is given by

$$u_t = -1.002uu_x + 0.0032u_{xx}$$

which shows great agreement with the ground truth. The trained "root-branch" network can accurately reproduce distinctive system responses even with limited measurements under 10% noise, giving a full-field ℓ_2 error of 0.65%, as shown in Fig. 7*B-D*.

2.3.2 Fitzhugh-Nagumo type of Reaction-Diffusion equations

We consider the Fitzhugh-Nagumo (FN) type reaction-diffusion system, in a 2D domain $\Omega = [0, 150] \times [0, 150]$ with periodic boundary conditions, whose governing equations are expressed by two coupled PDEs [10, 11]:

$$\begin{aligned} u_t &= \gamma_u \Delta u + u - u^3 - v + \alpha \\ v_t &= \gamma_v \Delta v + \beta(u - v) \end{aligned}$$

where u and v represent two interactive components/matters (e.g., biological), $\gamma_u = 1$ and $\gamma_v = 100$ are diffusion coefficients, $\alpha = 0.01$ and $\beta = 0.25$ are the coefficients for reaction terms, and Δ is the Laplacian operator. The FN equations are commonly used to describe biological neuron activities excited by external stimulus (α), which exhibit an activator-inhibitor system because one equation boosts the production of both components while the other equation dissipates their new growth. The

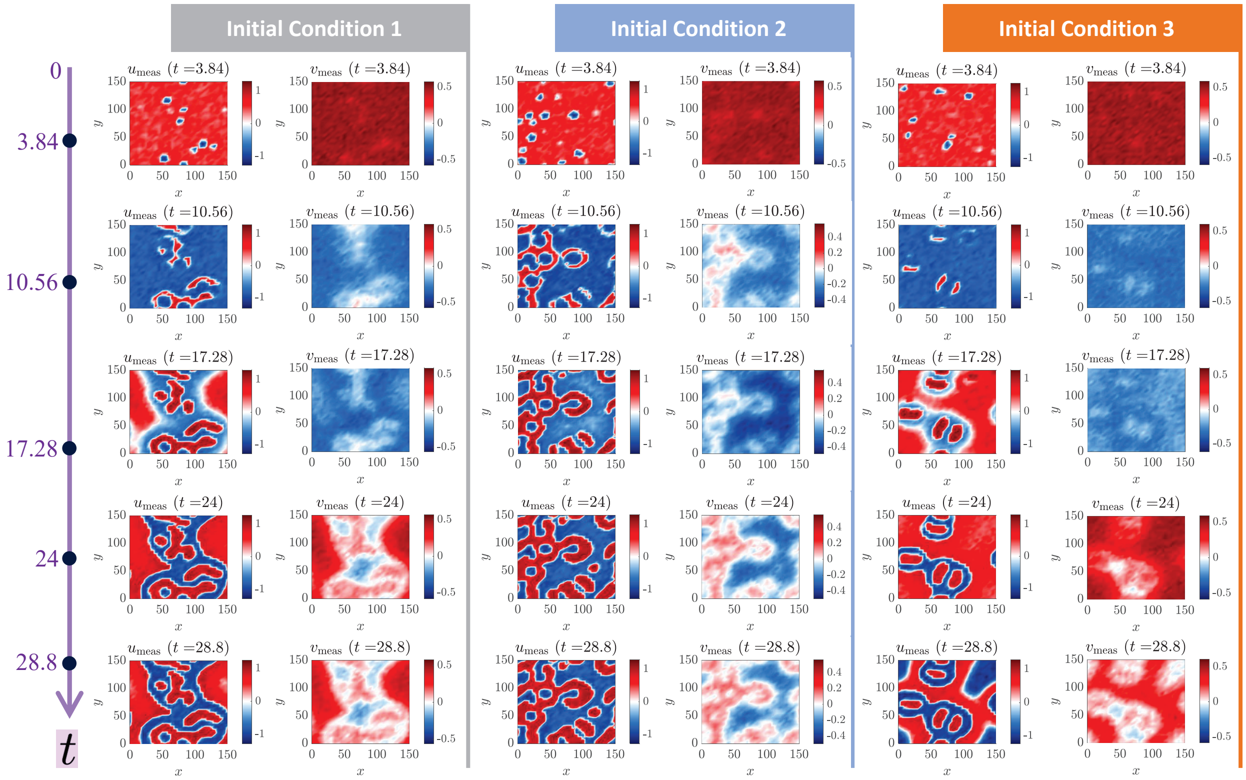

Figure 8: A few typical snapshots of low-resolution noisy measurements (10% noise) sampled from the system response under three different initial conditions (ICs) for discovering Fitzhugh-Nagumo equations. Note that the measurement data consists of 31 low-resolution noisy snapshots (with a grid size of 31 × 31) for each IC uniformly sampled within the time range of [0 28.8].

ground truth data is generated by the finite difference method ($dx = dy = 0.5$ and $dt = 0.0002$) for the time period of $[0, T] = [0, 36]$, with three random fields as initial conditions. Three measurement datasets are then generated, each of which consists of 31 low-resolution snapshots (projected into a 31×31 grid) uniformly down-sampled from full-field synthetic data during the period of $[7.18, 36]$ under a 10% noise condition (see Fig. 8). Similar to the previous example in Section 2.3.1, we design a "root-branch" DNN with three branches: the root net has 2 hidden layers of 60 nodes while each of the three branch nets has 3 hidden layers of 60 nodes, whose activation functions and initializations are the same as previous. We sample 5×10^4 spatiotemporal collocation points using Latin hypercube sampling [3] to construct the physics residuals.

We assume the diffusion terms (Δu and Δv) are known in the PDEs, whose coefficients (γ_u and γ_v) yet need to be identified. We employ the bounds to these two positive coefficients to speed up the convergence, namely, $\gamma_u \in [0, 5]$ and $\gamma_v \in [0, 150]$. We design 70 candidate functions, composed of up to third-order polynomials (including the constant term "1" as the zero order), derivatives $\{u_x, u_y, u_{xy}, v_x, v_y, v_{xy}\}$ and their mutual multiplication, to reconstruct the nonlinear reaction terms in the PDEs. Hence, the final library has 72 candidate terms, whose initial coefficients are randomly chosen between -1 and 1. To account for the small stimulus term (e.g., 0.01 in the first equation), we increase the sensitivity of the constant candidate "1" in the library by down-scaling its magnitude to the order of 10^{-5} and 5×10^{-4} for u and v equations respectively. Due to the memory issue, we run this case on a workstation with an NVIDIA Tesla V100 GPU card (32 GB). The training efforts include the pretraining stage with 2×10^3 Adam epochs and 2×10^4 L-BFGS epochs, 6

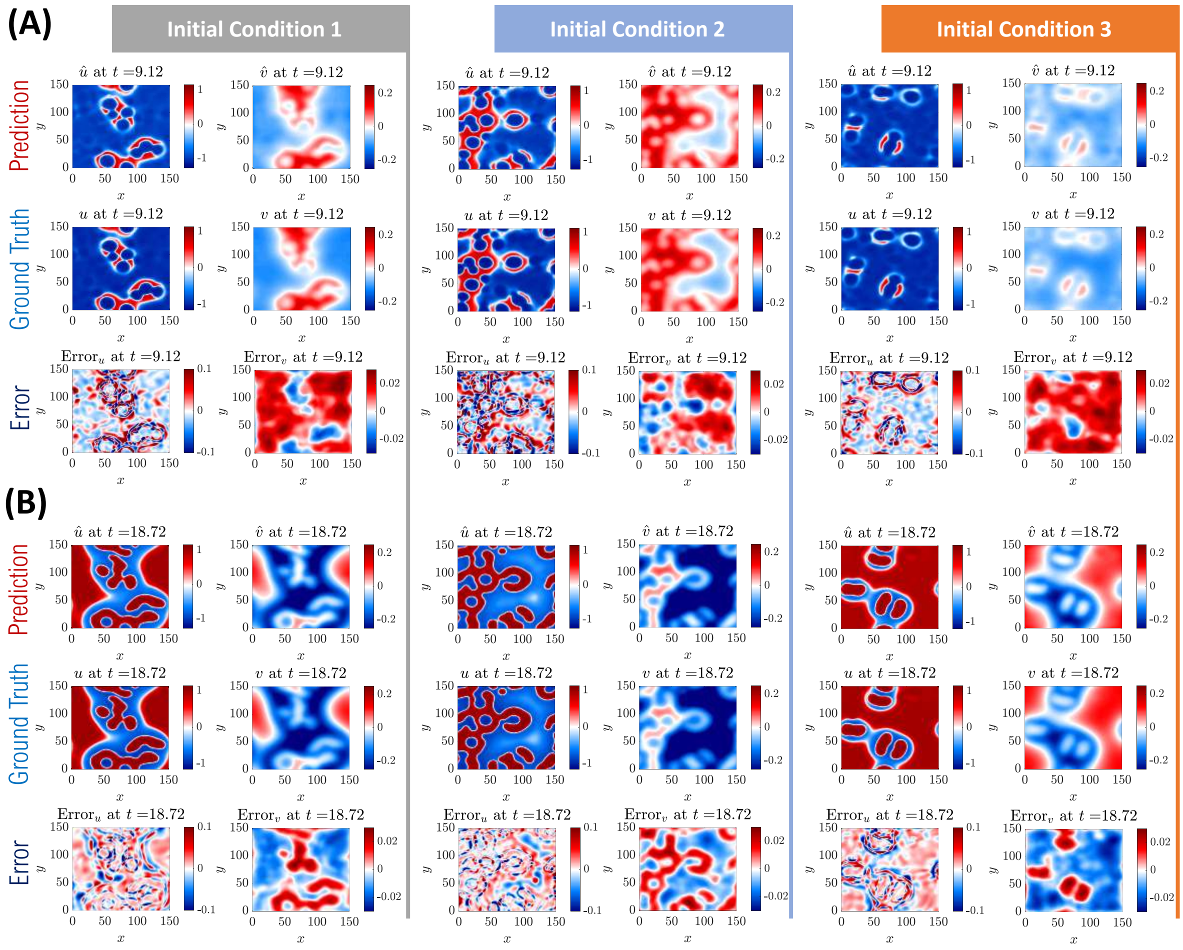

Figure 9: Discovery of the Fitzhugh-Nagumo equations based on measurements sampled under three initial conditions (ICs) with 10% noise: (*A*-*B*) Snapshots of predicted response, ground truth and error distributions for all three ICs at two unmeasured time instances ($t = 9.12$ and $t = 18.72$). The relative ℓ_2 error for the predicted full-field response (stacked u and v) is 5.02%.

ADO iterations, and an extra post-training with 2×10^4 Adam epochs and 2×10^4 L-BFGS epochs. In each ADO iteration, we use 1×10^3 Adam epochs in synergy with STRidge. To deal with the aforementioned bounds for γ_u and γ_v in an unconstrained optimization process, we set $\gamma_u = 5\sigma(\tilde{\gamma}_u)$ and $\gamma_v = 150\sigma(\tilde{\gamma}_v)$ and take $\{\tilde{\gamma}_u, \tilde{\gamma}_v\}$ as trainable variables, where $\sigma(\cdot)$ denotes the Sigmoid function. The discovered equations under 10 % noise is

$$u_t = 0.975\Delta u + 0.871u - 0.847u^3 - 0.924v + 0.010$$
$$v_t = 84.339\Delta v + 0.225u - 0.229v$$

It is seen that the form of the PDEs is precisely uncovered with all correct active terms (including the unknown external stimulus in the first equation). The corresponding identified coefficients are generally close to the ground truth (error of non-zero coefficients: $9.05 \pm 5.66\%$) except the diffusion coefficient for v (i.e., γ_v) which seems to be a less sensitive parameter according to our test. It should be noted that, given very scarce and noisy measurement datasets in this example, the "root-branch" DNN is faced with challenges to accurately model the solutions with sharp propagating fronts (see Fig. 9*C*-*D*). The less accurate solution approximation by DNN then affects the discovery precision. This issue can be naturally alleviated by increasing the spatiotemporal measurement resolution

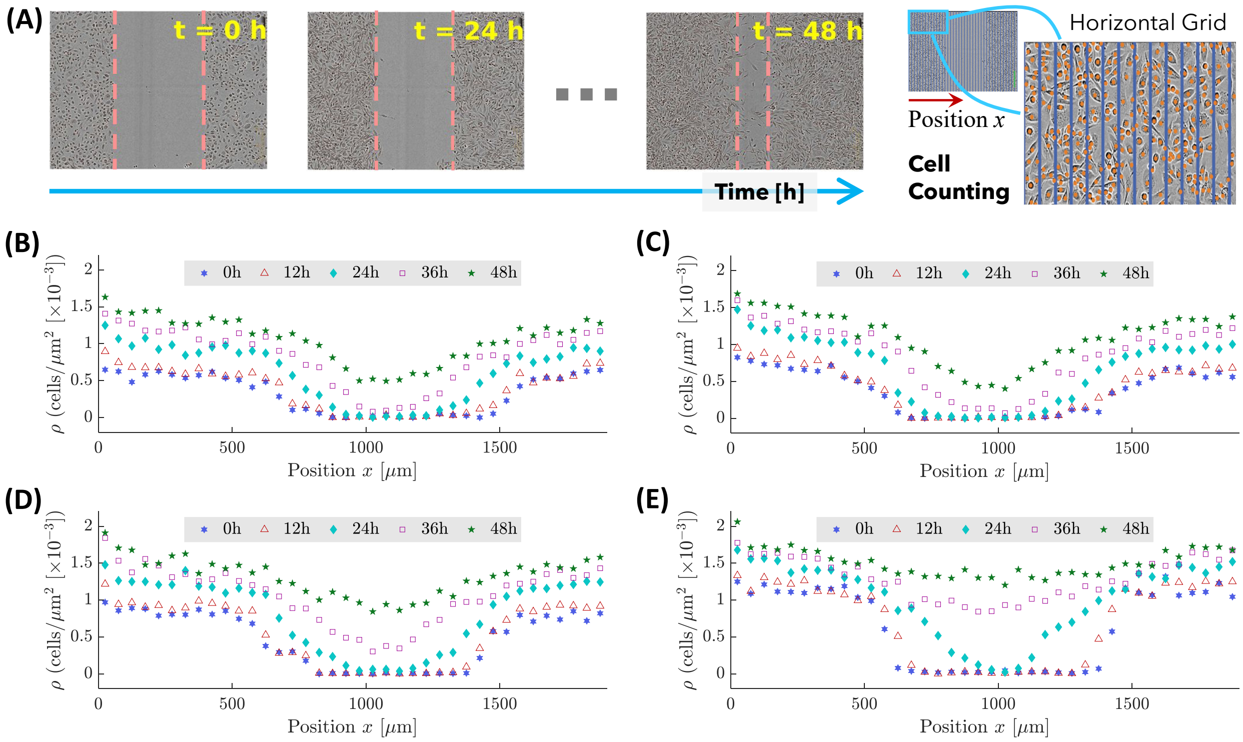

Figure 10: Measurement datasets of cell densities, ρ, based on scratch assays [12]. (A) Example scratch assay imaging of 16,000 cells in the test well with a width of 1,900 μm (the images are reproduced from Jin *et al.* [12]). The images are taken at different time instants (0h, 12h, 24h, 36h, 48h). The dashed lines show the approximate location of the positions of the leading edge. These images are then evenly divided into 38 segments (50 μm each) horizontally, where the cells are counted in each segment to determine the horizontal cell densities. (B-E) the cell densities at different time instants for 14,000, 16,000, 18,000 and 20,000 cells, respectively.

(even still under fairly large noise pollution, e.g., 10%). Nevertheless, the exact form of the PDEs is successfully discovered in this challenging example, which is deemed more important since the coefficients can be further tuned/calibrated when additional data arrives. The evolution of the PDE coefficients corresponding to 72 candidate functions for $\hat{u}$ and $\hat{v}$ is illustrated in Fig. 9A and B, respectively. Note that, for visualization purpose, we re-scale the identified coefficients of the constant stimulus term "1" in the u-equation by multiplying 100 in Fig. 9A and the diffusion term Δv in the v-equation by dividing 50 in Fig. 9B. The trained network is finally used to predict the full-field responses under three I/BCs (see the snapshots in Fig. 9C-D at two unmeasured time instants). The stacked full-field ℓ_2 error is 5.02%.

2.4 Experimental discovery of cell migration and proliferation

In this example, we consider to discover a biological system based on scratch assay experiments [12] investigating the cell migration and proliferation process. The 1D cell density distributions at different time instants (0h, 12h, 24h, 36h, 48h) were extracted and simplified from high-resolution imaging via image segmentation and cell counting (see Fig. 10). A series of assays were performed under different initial cell densities (e.g., the total number of cells spans from 10,000 to 20,000 following the designated initial distribution in the test well. More detailed description of the experiment setup and datasets can be found in [12].

Our objective herein is to uncover a parsimonious PDE for modeling the dynamics of cell density

$\rho(x, t)$. Here, we consider four scenarios with the initial number of cells ranging from 14,000, 16,000, 18,000 to 20,000. We take the mean of the test data from three identically-prepared experimental replicates for each scenario for PDE discovery. Each mean dataset has a total of 38×5 measurement points for five time instances. To improve the optimization condition, all measurements are upscaled by 1000 before being used in network training. Given our prior knowledge that the cell dynamics can be described by a diffusion (migration) and reaction (proliferation) process, we assume the PDE holds the form of $\rho_t = \gamma\rho_{xx} + \mathcal{F}(\rho)$, where γ is the unknown diffusion coefficient and $\mathcal{F}$ denotes the underlying nonlinear reaction functional. We use 8 additional candidate terms (e.g., $\{1, \rho, \rho^2, \rho^3, \rho_x, \rho\rho_x, \rho^2\rho_x, \rho^3\rho_x\}$) to reconstruct $\mathcal{F}$, whose coefficients are sparse. Hence, the total number of trainable coefficients remains 9 (e.g., $\mathbf{\Lambda} \in \mathbb{R}^{9\times1}$), whose initial values are randomly sampled from $[-1, 1]$. We believe incorporating our domain-specific prior knowledge is reasonable and should be encouraged in interpretable model discovery, which could help improve our solution confidence when available data is very sparse and noisy (e.g., in this example).

We sample 1×10^4 collocation pairs using Latin hypercube sampling [3] in the spatiotemporal domain of $\Omega \times [0, T] = [0, 1900]\mu\text{m} \times [0, 48]\text{h}$. The DNN has 3 hidden layers of 30 nodes activated by by the tanh function (see Fig. 1 in the Main Text). Considering that the cell density is constantly positive, we impose a *softplus* function (e.g., $\ln(1+e^z)$) in the output layer to curb the final output of ρ. To account for potential large magnitude variation of the candidate term coefficients, we apply the tanh function to squash magnitude gaps. Specifically, we set $\gamma = 1000\tanh(\tilde{\gamma})$ and $\boldsymbol{\lambda} = 50\tanh(\tilde{\boldsymbol{\lambda}})$, where $\tilde{\gamma}$ and $\tilde{\boldsymbol{\lambda}}$ are trainable "proxies" for diffusion coefficient γ and other 8 coefficients $\boldsymbol{\lambda}$ (note: $\mathbf{\Lambda} = \{\gamma, \boldsymbol{\lambda}\} \in \mathbb{R}^{9\times1}$). The training efforts include the pretraining stage with 8×10^3 Adam epochs and up to 8×10^3 L-BFGS epochs, 6 ADO iterations, and an extra post-training with 2×10^4 Adam epochs and up to 2×10^4 L-BFGS epochs. In each ADO iteration, we use 1×10^3 Adam epochs in synergy with STRidge. The discovered underlying PDEs under different initial cell states are given as follows:

$$\begin{aligned}
\text{14k cells:}\quad \rho_t &= 530.39\rho_{xx} + 0.066\rho - 46.42\rho^2\\
\text{16k cells:}\quad \rho_t &= 484.74\rho_{xx} + 0.065\rho - 43.15\rho^2\\
\text{18k cells:}\quad \rho_t &= 636.68\rho_{xx} + 0.070\rho - 45.48\rho^2\\
\text{20k cells:}\quad \rho_t &= 982.26\rho_{xx} + 0.078\rho - 47.65\rho^2
\end{aligned}$$

which share a unified form of $\rho_t = \gamma\rho_{xx} + \lambda_1\rho + \lambda_2\rho^2$ which exactly matches the famous Fisher-Kolmogorov model [13, 14]. The rates of migration (diffusion) and proliferation (reaction) generally increase along with the number of cells, as seen from the identified coefficients. Fig, 11*A*-*D* depict the learned cell density profiles by the trained DNN, which capture the critical patterns of the measurement while showing little evidence of overfitting. With the discovered PDEs, we simulate/predict the evolution of cell densities at different time instants (12h, 24h, 36h and 48h) presented in Fig. 11*E*-*H*, where the measurement at 0h is used as the initial condition while $\rho_x(x = 0, t) = \rho_x(x = 1900, t) = 0$ is employed as the Neumann boundary condition. The satisfactory agreement between the prediction and the measurement provides a clear validation of our discovered PDEs.

3 Discussion

In this section, we discuss several other features, influence factors and limitations of the proposed PINN-SR method for data-driven discovery of PDEs, and highlight the potential future work.

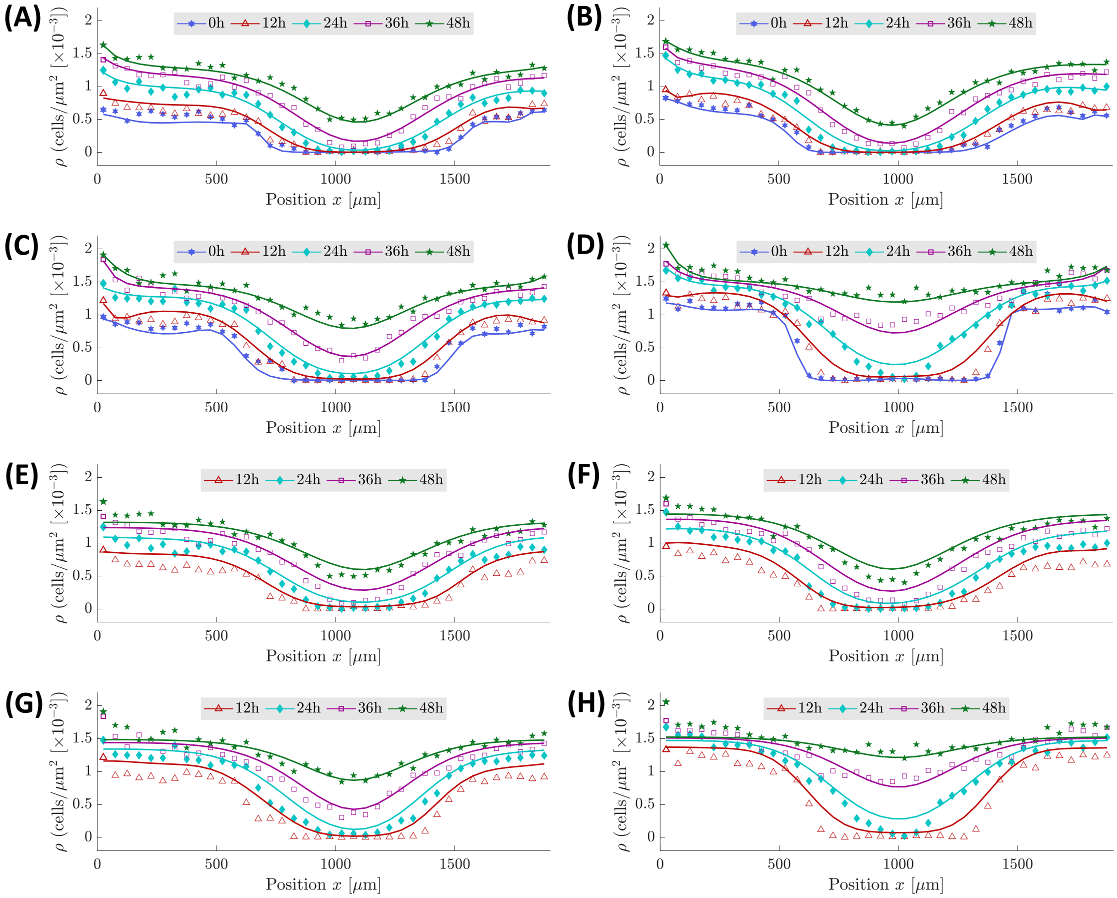

Figure 11: Experimental discovery of cell migration and proliferation: (A)-(D) Predicted cell densities (represented by solid curves) by the trained DNNs in comparison with the measurement data (denoted by markers) for 14,000, 16,000, 18,000 and 20,000 cells, respectively. (E)-(H) Simulated cell densities, represented by solid curves, at different time instants based on the discovered PDEs for 14,000, 16,000, 18,000 and 20,000 cells, respectively, where the measurement at 0h is used as the initial condition while $\rho_x(x = 0, t) = \rho_x(x = 1900, t) = 0$ is employed as the Neumann boundary condition. The simulation result is represented by solid curves while the markers denote the measurement data.

3.1 Simultaneous identification of unknown source

In practical applications, the physical system might be subjected to spatiotemporal source input ($\mathbf{p}$) which is unknown and can be only sparsely measured. When discovering the underlying governing equation for such a system, the source should be considered and reconstructed concurrently. In this case, we incorporate the source candidate functions into the library $\boldsymbol{\phi}$ for simultaneous discovery of the PDE and reconstruction of the unknown source. Thus, the sparse representation of the PDE(s) can be written as

$$\mathbf{u}_t = [\boldsymbol{\phi}^u \ \boldsymbol{\phi}^p][\boldsymbol{\Lambda}^u \ \boldsymbol{\Lambda}^p]^T$$

where $\boldsymbol{\phi}^u$ and $\boldsymbol{\phi}^p$ denote the libraries of candidate functions, while $\boldsymbol{\Lambda}^u$ and $\boldsymbol{\Lambda}^p$ are the corresponding sparse coefficients, for the field variable $\mathbf{u}$ and the source $\mathbf{p}$, respectively. To demonstrate this concept, we test the Burgers' equation driven by a source term, expressed as

$$u_t + uu_x - 0.1u_{xx} = \sin(x)\sin(t).$$

To generate the solution, the problem domain is meshed into 201 spatial grid points for $x \in [-5, 5]$ and 101 time steps for $t \in [0, 10]$. We use 20 fixed sensors randomly selected from the spatial grid points to record the wave response (u) for 50 time steps, polluted with 10% noise. Note that the source is not measured and regarded as unknown.

The following libraries of candidate function are used to reconstruct the PDE and the source:

$$\boldsymbol{\phi}^u = \{1, u, u^2, u^3, u_x, uu_x, u^2u_x, u^3u_x, u_{xx}, uu_{xx}, u^2u_{xx}, u^3u_{xx}, u_{xxx}, uu_{xxx}, u^2u_{xxx}, u^3u_{xxx}\}$$

$$\boldsymbol{\phi}^p = \{a, b, c, d, a^2, b^2, c^2, d^2, ac, ab, ad, bc, bd, cd\}$$

where $a = \sin(t)$, $b = \sin(x)$, $c = \cos(t)$ and $d = \cos(x)$. The hyperparameters for the PINN-SR network are similar those used in the previous Burgers' example. The pre-training takes up to 15×10^3 epochs of Adam and about 1×10^3 epochs of L-BFGS, followed by 20 ADO iterations. In each ADO iteration, we use the Adam optimizer with 1×10^3 epochs and the L-BFGS with up to 1×10^3 epochs to train the DNN for each alternation within STRidge. The discovered PDE along with the uncovered source term is given by

$$u_t + 1.002uu_x - 0.088u_{xx} = 0.995 \sin(x) \sin(t).$$

It can be seen by comparing the above two equations that both the sparse terms and the corresponding coefficients are accurately identified, despite only scarce and noisy measurement of the system response is supplied. Although only 4.9% subsampled responses are measured while the source information is completely unknown, the PINN-SR approach can reasonably well extrapolate the full-field solution with a ℓ_2 error of 13.8% (see Fig. 12A). The major errors are mostly distributed close to the boundaries due to scarce training data. Fig. 12B shows the comparison of spatial and temporal snapshots between the predicted and the exact solutions which match well with each other.

Nevertheless, if the source is very complex with its general expression or form completely unknown, distinct challenges arise when designing the source library of candidate functions $\boldsymbol{\phi}^p$. This may require an extraordinarily large-space library to retain diversifying representations, and thus pose additional computational complexity for accurate discovery of the PDEs. In some specific cases, the unknown source term can probably be approximated by the combination of continuous basis functions such as the Fourier series and the Lagrange polynomials, instead of finding its closed form. These open questions will be addressed in our future work.

3.2 Effect of missing candidate terms

Since the majority of well-known first-order PDEs w.r.t. time can be represented by linear combination of several active linear/nonlinear terms, we try to include as many as possible commonly seen terms following polynomial basis. Failing to include essential candidate functions will lead to false-positive discovery of parsimonious closed form of PDEs, despite that a "best-of-fit" form can be found. To investigate the effect of missing candidate term(s), we consider a special case where the actual candidate uw_x is intentionally ignored from the original library for discovery of the NS equation, leading to an inadequate library with 59 candidate functions. All hyper-parameters and training efforts are the same as those in Section 2.1.4. The discovered equation is given by:

$$w_t = -0.253w_x + 0.008w_{yy} + 0.035uw_{xx} - 0.782u^2w_x - 0.026u^2w_{xx} - 0.616vw_y - 0.155v^2w_x - 0.526uvw_y$$

It can be seen that the discovered equation is less parsimonious and has much more terms compared with the ground truth. The predicted full-field error for w increases from 2.58% (see Section 2.1.4)

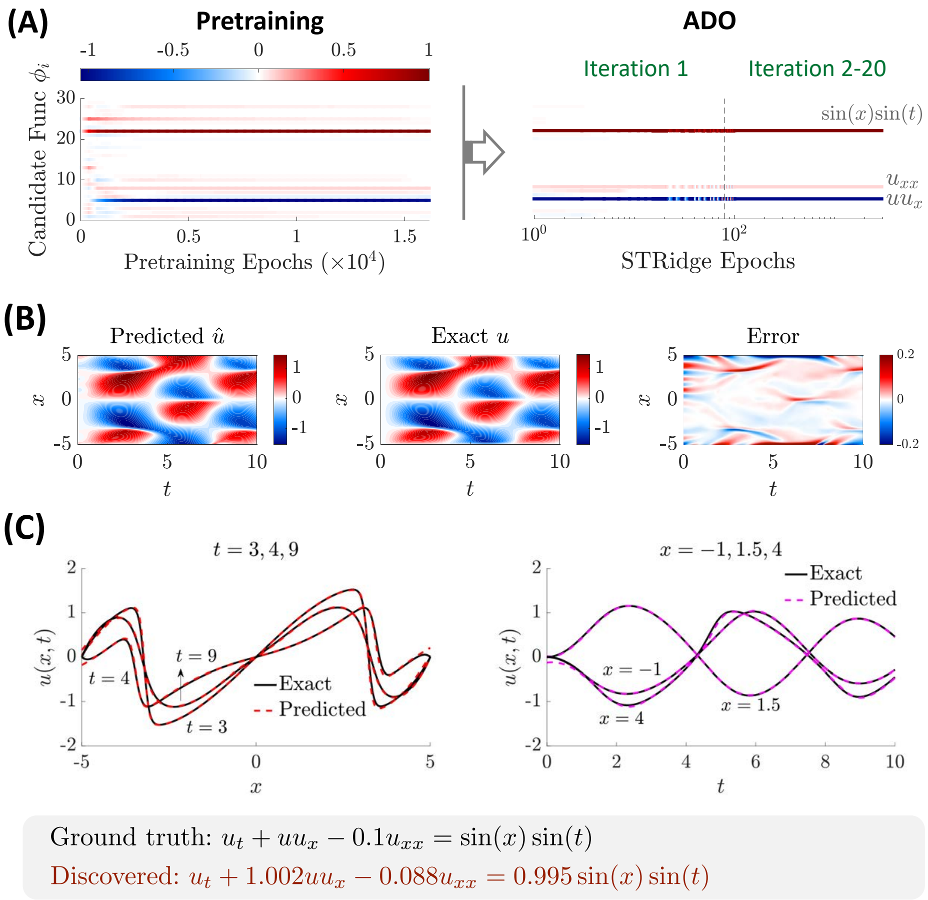

Figure 12: Discovery of Burgers' equation and source term for measurement data with 10% noise: (A) Evolution of the sparse coefficients $\mathbf{\Lambda} \in \mathbb{R}^{30\times 1}$ for 30 candidate functions $\boldsymbol{\phi} \in \mathbb{R}^{1\times 30}$ used to form the PDE and the unknown source term, where the color represents the coefficient value. The predicted response in comparison with the exact solution with the prediction error. (B) Comparison of spatial and temporal snapshots between the predicted and the exact solutions. The relative full-field ℓ_2 error of the prediction is 13.8%. The major errors are mostly distributed close to the boundaries due to scarce training data.

to 5.25%. We observe that given an insufficient library, the discovery is likely to result in a non-parsimonious equation, where redundant terms are found to replenish the lost pattern of uw_x, and consequently yields less accurate response prediction.

3.3 Noisy measurements and collocation points

The total loss function is evaluated on the measurement data (for $\mathcal{L}_d$) and the collocation points (for $\mathcal{L}_p$). Therefore, the availability of noisy measurement data and the number of collocation points sampled from the spatiotemporal space will affect the convergence of the PINN-SR model and thus the PDE discovery accuracy. We herein study the sensitivity of PINN-SR to these factors in the context of discovery accuracy based on the Burgers' equation example. In particular, we use the relative ℓ_2-norm error to reflect the global accuracy of of the identified sparse coefficients, defined as $e_\lambda = ||\hat{\mathbf{\Lambda}} - \mathbf{\Lambda}_{\text{true}}||_2/||\mathbf{\Lambda}_{\text{true}}||_2$ where $\hat{\mathbf{\Lambda}}$ denotes the identified coefficients and $\mathbf{\Lambda}_{\text{true}}$ is the ground truth. Fig. 13 shows the error metrics for discovering the Burgers' equation under different quantities of measurement points and collocation points and noise levels. Increasing the number of data points in the measurement (e.g., recorded by more sensors) can well compensate the noise

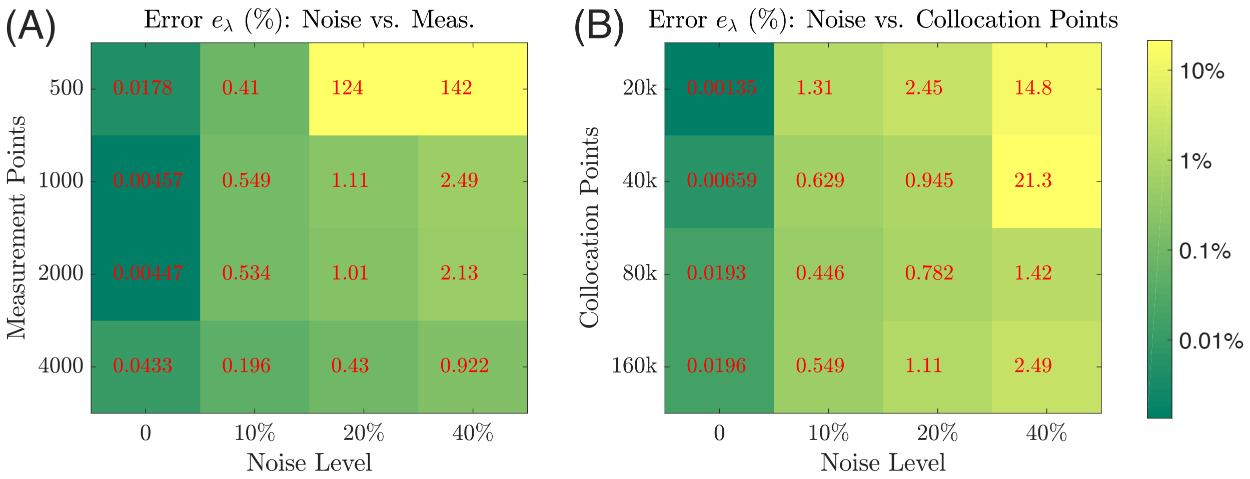

Figure 13: Error e_λ for discovery of Burgers' equation under different measurement points, collocation points and noise levels. Numbers in each cell denote the percentage error of e_λ for a specific condition, which is the relative ℓ_2 norm error between the identified coefficients $\mathbf{\Lambda}$ and the ground truth $\mathbf{\Lambda}_{\text{true}}$. The color also indicates the error level. The collocation points are fixed at 1.6×10^5 in (A), while the measurement points are always 1×10^3 in (B).

Table 2: On the extrapolation (generalization) ability of PINN-SR

Case	Meas. points	Collocation points	Noise level	Training error	Validation error	Full-field error	ℓ_2 error of $\mathbf{\Lambda}$
1	1.5×10^3	8×10^4	0	0.03%	0.04%	2.41%	0.02%
2	1.5×10^3	8×10^4	10%	5.73%	5.95%	4.50%	0.79%
3	1.5×10^3	0	0	58.79%	60.19%	144.99%	142.66%
4	3×10^3	0	0	0.10%	0.10%	14.36%	0.38%

Note: The training error, validation error and full-field error are calculated in the form of $\|\hat{\mathbf{u}} - \mathbf{u}_{\text{true}}\|_2 / \|\mathbf{u}_{\text{true}}\|_2$, where $\hat{\mathbf{u}}$ denotes the DNN-predicted response and $\mathbf{u}_{\text{true}}$ is the reference ground truth solution.

effect as shown in Fig. 13A (the number of collocation points is fixed at 1.6×10^5), which agrees with our common sense. Although optimal sensor placement might alleviate the need of large datasets [15], this is out of the scope of this work. The use of more collocation points can mitigate the noise effect and improve the discovery accuracy as illustrated in Fig. 13B (the number of measurement points is fixed at 1×10^3). For this specific case, 2×10^4 (or more) collocation points are able to maintain a satisfactory discovery accuracy for measurements under noise corruption at a realistic level (e.g., $\leqslant 20\%$). When the data are sampled under a very noisy condition (e.g., 40% noise level), the proposed method is still robust if a larger number of collocation points are used (e.g., $\geqslant 8 \times 10^4$).

It is noteworthy that the collocation points require no correlation with the measurement data. In particular, we use the Sobol sequence [16] (or Latin hypercube sampling [3] which is also applicable) to simulate a finer uniform partitions of the problem domain, making the random sampling of collocation points more representative. Intuitively, the more the collocation points are used, the more generalizable the trained network will be and the more accurate the discovered PDE is. However, a large number of collocation points also impose heavy computational burden, limited by hardware resources. A fairly large amount of collocation samples (e.g., on the order of magnitude of $> 10^4$), comparable to the complexity and dimension of the discovery problem, are suggested in practical applications meanwhile considering the memory of the computing machine.

We further conduct a comparative study on the role of collocation points and seek for numerical understanding of how much they can help for extrapolation. Taking the Burgers' equation for instance, we define an enclosed area, part of the full-field response, as shown in Fig. 14, and sample the measurement data only within such an area. We intend to reconstruct the full-field response

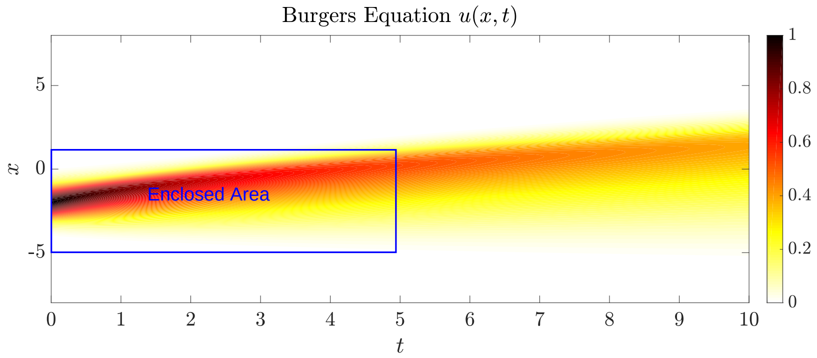

Figure 14: Parametric study on the effect of collocation points for discovering the Burgers' equation. The measurements are only taken from the enclosed area, while the collocation points are sampled across the full field.

beyond the enclosed area and discover the PDE taking advantage of collocation points. More specifically, the enclosed area is meshed by 100×50 spatiotemporal points. We take 30 randomly selected spatial locations as fixed sensors recording the dynamic response of the system, resulting in 1.5×10^3 data points. Additionally, we sample 8×10^4 collocation points from the full spatiotemporal field for evaluating the residual physics loss during model training. Four cases are considered to demonstrate the function of collocation points with measurements sampled in the enclosed area (see Table 2).

Provided with clean measurements from the enclosed area and global collocation points, PINN-SR does an impressive job on both full-field response reconstruction and sparse coefficients identification (see Case 1 in Table 2). When the measurements become noisy (e.g., 10% noise level), despite the response prediction errors increase, the PDE can still be accurately discovered (see Case 2 in Table 2). If we consider removing all collocation points and only train the network with clean measurements, the response prediction errors (even during the training and validation stage) all remain over 50%, meanwhile the PDE is also completely misidentified (see Case 3 in Table 2). Once we double the clean measurement points to 3×10^3, the trained DNN has strong interpolation and discovery abilities; however, the trained network does a poor job in extrapolating the full-field response (see Case 4 in Table 2). Concluding from this parametric test, we can see that the collocation points can render PINN-SR tolerable to scarce and noisy measurements, making the DNN generalizable.

3.4 On the effect of non-Gaussian noise

To explore the effect of non-Gaussian noise, we test our algorithm on discovery of the FN equations based on a series of low-resolution snapshots blurred/smoothed by a 2D Gaussian filter (e.g., the resulting noise in the measurement data is always positive). In particular, the original high-resolution data for the case of IC3 (see Section 2.3.2) is firstly smoothed by two 2D Gaussian filters, of standard deviation 2 and 12.5 and of spatial size 9×9 and 51×51 for u and v, respectively. Afterwards, we uniformly sub-sample 31 low-resolution snapshots (projected into a 31×31 spatial grid) from the blurred dataset and take them as our measurement data. The rest algorithm settings are the same as those in Section 2.3.2. The discovered equations are given as follows:

$$\begin{aligned} u_t &= 1.164\Delta u + 0.911u - 0.912u^3 - 0.939v + 0.011 \\ v_t &= 102.648\Delta v + 0.245u - 0.244v \end{aligned} \tag{1}$$

It is seen that the discovered PDEs bear high resemblance to the ground truth. Fig. 15 displays

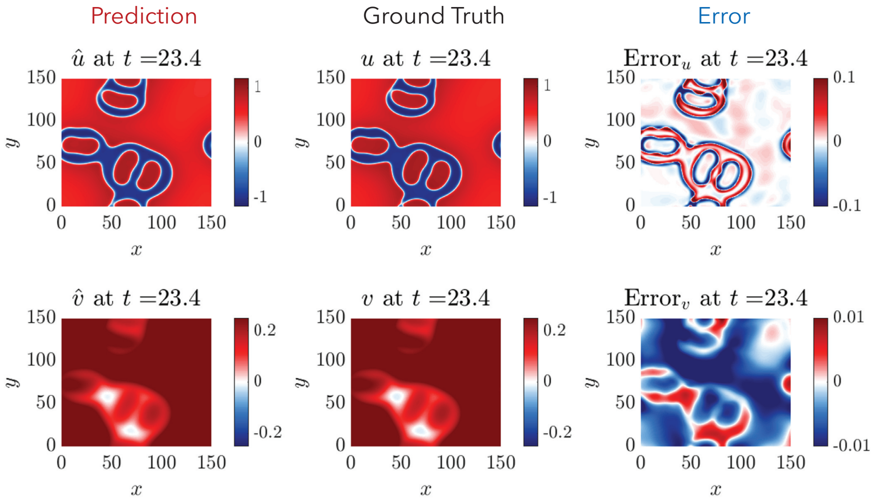

Figure 15: Discovery of Fitzhugh-Nagumo equations based on dataset corrupted by non-Gaussian-type noise: snapshots of the predicted response, ground truth and error at an unmeasured time instance ($t = 23.4$).

the evolution of 72 library coefficients $\mathbf{\Lambda}$. We further predict the full field response of u and v at a high-resolution grid 151×151 for 241 snapshots uniformly distributed in $[0, T] = [0, 28.8]$. The predicted solution field at one unmeasured time instance $t = 23.4$ is visualized in Fig. 15 which shows the sporadic evolution of this system with sharp propagating fronts. The error contours at the same time instance mainly appear in the propagation fronts. The overall error for all 241 predicted high-resolution images is about 3.71%. We can conclude that the proposed method is also capable of handling non-Gaussian-type noises.

3.5 Optimal sensor placement

In fact, optimal placement of sensors is very important in inverse problems, which could provide informative datasets facilitating discovery. Ideally, sensors should be efficiently installed in the most representative region of the system with sharp gradients (such as Fig. 14), even though such a deployment will require a stronger foresight of how the system will behave. There are active learning strategies similar to [17] that can place new sensors in an online manner to minimize large physics residue in some local areas. Nevertheless, such an investigation is out of the scope of this work. In our present study, since we assume that we have little prior knowledge on the system response, it is infeasible to proactively deploy sensors in specific regions (e.g., the shock developing regions). In particular, when multiple I/BCs are considered, these regions might vary case by case. For instance, in the example of Burgers' equation with shock behavior (see Section 2.3.1), the shock developing regions are very different. Therefore, we choose to randomly place the sensors in space to cover various possibility.

3.6 Convergence history

As an example, the total loss history for the NS example (see Section 2.1.4) is shown in Fig. 16, where the loss decreases remarkably during pre-training when both DNN's parameters and the PDE candidate term coefficients are trained to model the paramount patterns revealed by the noisy data (e.g., 10% noise). The switch between Adam and L-BFGS occurs at the Epoch 5000. In the ADO stage, the parsimonious equation structure is adaptively extracted while the model carefully maintains prediction ability, where in consequence the loss doesn't drop too much. The

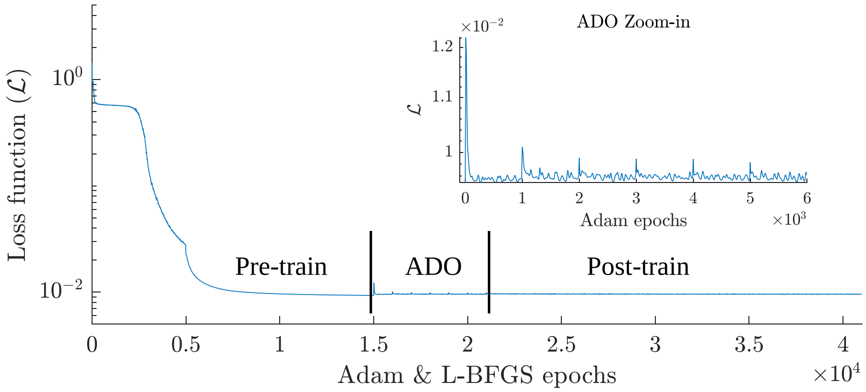

Figure 16: The history of the total loss for Navier-Stokes vorticity equation. The loss decreases remarkably during pre-training, where both DNN's weights and biases and library coefficients learn the paramount patterns in the data. In ADO stage, the parsimonious equation structure is adaptively extracted while the model carefully maintains prediction ability, in consequence the loss doesn't drop too much. The loss mildly declines in the end due to the refinement of DNN parameters' and non-zero equation coefficients in particular.

Table 3: Results for different settings of network size in Burgers' equation.

Model	No. of hidden layers	No. of nodes	Full-field ℓ_2 error	Equation coefficient error	Time
1	4	20	0.19%	0.35±0.28%	381 s
2	4	40	0.89%	0.53±0.37%	594 s
3	6	20	0.49%	0.36±0.12%	583 s
4	6	40	0.88%	0.72±0.21%	1055 s
5	8	20	0.51%	0.36±0.33%	553 s
6	8	40	1.12%	0.54±0.31%	1034 s

jump at the beginning of ADO is due to the update and pruning of $\hat{\mathbf{\Lambda}}$ in the STRidge. The loss mildly decreases in the post-training stage due to the refinement of DNN parameters' and non-zero equation coefficients.

3.7 A parametric study on network size

The network configurations are adopted based on common practices in popular PINN literature [18–22] and our empirical observations. We found that networks with 4-8 hidden layers, each having 20-60 nodes, are generally sufficient for accurate modeling the system behaviors in our discovery problems, depending on the problem complexity. To further show the effect of the network configuration, we perform a parametric studies in one typical example (e.g., the Burgers' equation) on the number of hidden layers {4, 6, 8} with the number of nodes {20, 40} in each hidden layer. In all cases, we use the same hyper-parameters as those in 2.1.1 except that the measurements are noise-free here. The analyses are performed on a workstation with 28 Intel Core i9-7940X CPUs and 2 NVIDIA GTX 1080Ti GPU cards. Results of 6 different network configurations are shown in Table 3, where it is clear that as long as the layers and nodes are sufficient for solution approximation, it ensures a successful discovery. However, a deeper or wider network requires more training effort and results in longer computational time. Furthermore, a bigger network is more susceptible to overfitting, as the full-field prediction error increases with the growth of network width.

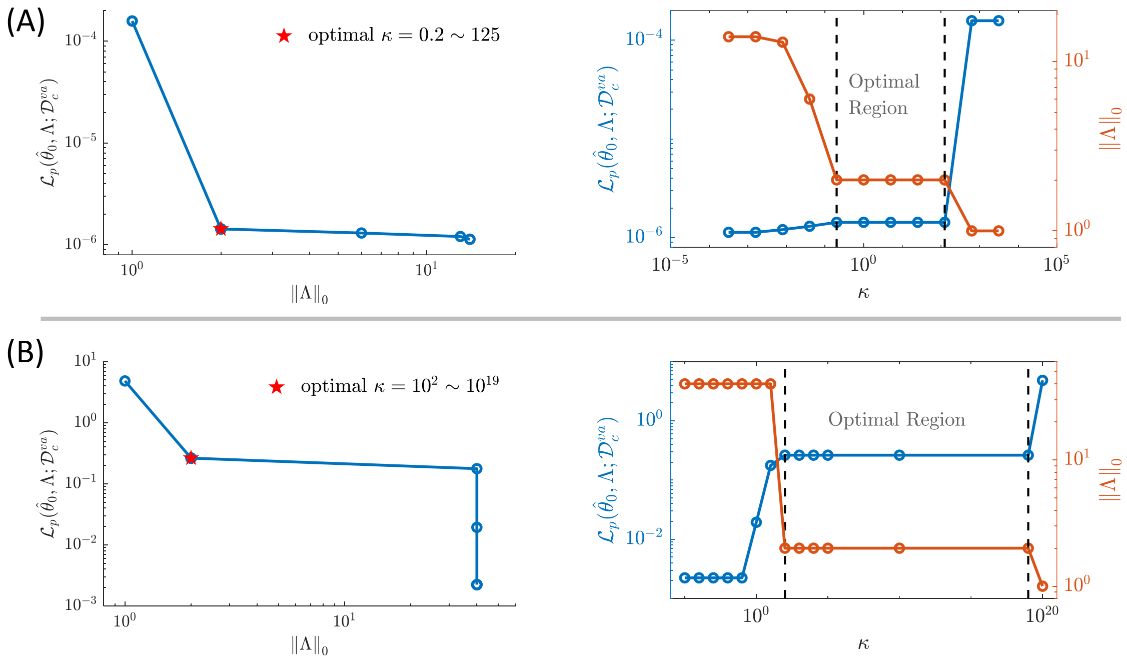

Figure 17: Pareto front analysis for determining the value of β balance model accuracy and sparsity/parsimony for datasets with 10% noise: (A) Burgers' equation and (B) nonlinear Schrödinger equation. The proper value of β should be selected in the Optimal Region as shown in the plots. For example, we select $\kappa = 1$ and $\kappa = 100$ for discovery of the Burgers and Schrödinger equations, respectively, as shown in Table 4. Note that $\beta = \kappa \mathcal{L}_p(\hat{\boldsymbol{\theta}}_0, \hat{\boldsymbol{\Lambda}}_0; \mathcal{D}_c^{va})$.

3.8 List of hyper-parameters used in the examples

Following the consistent criteria discussed in Main Text Method and Section 1.1, the list of hyper-parameters used in the above examples is summarized in Table 4. Here, we illustrate how β is selected based on Pareto front analysis. In particular, we take the Burgers' and nonlinear Schrödinger equations as an example. We firstly construct the sparse regression problem solved by STRidge, where $\dot{\mathbf{U}}$ and $\boldsymbol{\Phi}$ are evaluated based on the pre-trained DNN (with the trained network parameters denoted by $\boldsymbol{\theta}_0$). A grid search for β is then performed by running only the first (or two) ADO iterations to obtain the graphical representation of the Pareto set (e.g., $\mathcal{L}_p(\boldsymbol{\theta}_0, \boldsymbol{\Lambda}; \mathcal{D}_c)$ vs. $\|\boldsymbol{\Lambda}\|_0$). The optimal range of β can then be determined. Fig. 17 summarizes the analysis results.

3.9 Other limitations

We also observe some other limitations of our proposed method in its current version. For example, we failed to discover the Gray-Scott reaction-diffusion equation [23] (with several false-positives) whose PDE coefficients have orders of magnitude difference (e.g., diffusion coefficient 10^{-5} vs. reaction term coefficient 1). While such a magnitude difference can be solved by applying activation functions to equation coefficients (e.g. the FN case and the cell case), such a treatment requires extra prior knowledge. In addition, there are other factors that could bring challenges to our current framework, such as extremely low-quality measurements and pathology in PINN's gradient [21], which will be investigated in our future studies.

Table 4: The hyper-parameters used in the examples.

Example		$\alpha^{\mathbf{a}}$			γ	ADO				
		r_σ	Pre-training	ADO & Post-training		$\beta^{\mathbf{b}}$	ADO Iteration	Adam Epochs	STRidge Cycles	$\Delta\delta^{\mathbf{c}}$
Single Dataset	Burgers'	1.4	1	2	1E-7	$\mathcal{L}_p(\hat{\boldsymbol{\theta}}_0, \hat{\boldsymbol{\Lambda}}_0; \mathcal{D}_c^{va})$	6	1000	100	1
	KS	19.4	1	10	1E-7	$\mathcal{L}_p(\hat{\boldsymbol{\theta}}_0, \hat{\boldsymbol{\Lambda}}_0; \mathcal{D}_c^{va})$	6	1000	100	1
	Schrödinger	0.05	0.1	0.5	1E-7	$100\mathcal{L}_p(\hat{\boldsymbol{\theta}}_0, \hat{\boldsymbol{\Lambda}}_0; \mathcal{D}_c^{va})$	6	1000	100	100
	NS	1.7	1	2	1E-7	$\mathcal{L}_p(\hat{\boldsymbol{\theta}}_0, \hat{\boldsymbol{\Lambda}}_0; \mathcal{D}_c^{va})$	6	1000	100	1
	$\lambda - \omega$ RD	1.4	10	10	1E-7	$\mathcal{L}_p(\hat{\boldsymbol{\theta}}_0, \hat{\boldsymbol{\Lambda}}_0; \mathcal{D}_c^{va})$	6	1000	100	1
Multiple Datasets	Burgers'	0.02	0.01	0.1	1E-7	$\mathcal{L}_p(\hat{\boldsymbol{\theta}}_0, \hat{\boldsymbol{\Lambda}}_0; \mathcal{D}_c^{va})$	6	1000	100	1
	FN RD	17.2	1	10	1E-7	$\mathcal{L}_p(\hat{\boldsymbol{\theta}}_0, \hat{\boldsymbol{\Lambda}}_0; \mathcal{D}_c^{va})$	6	1000	100	1
Experimental	Cell	2.2E3	200	2.2E3	1E-7	$\mathcal{L}_p(\hat{\boldsymbol{\theta}}_0, \hat{\boldsymbol{\Lambda}}_0; \mathcal{D}_c^{va})$	6	1000	100	1

Note: **a.** The values of r_σ were estimated based on the measurement data (e.g., with 10% noise) and serve as a reference for determining α in ADO/post-training. In pre-training, we generally reduce the value of α to relax the physics constraint. A special case is the λ-ω equations, where **u** and **v** in the spiral dataset bear high resemblance, which require a larger α value. **b.** To balance the physics residue and the equation sparsity, the initial value for β can be set as the pre-trained physics loss $\mathcal{L}_p(\hat{\boldsymbol{\theta}}_0, \hat{\boldsymbol{\Lambda}}_0; \mathcal{D}_c^{va})$. However, a Pareto front analysis is typically required for determining this critical hyper-parameter. **c.** Sparsity threshold increment $\Delta\delta$ is usually set to 1, since STRidge can adaptively determine a normalized and optimal threshold except the Schrödinger cases. The Schrödinger equation is a complex-value system, therefore a larger initial $\Delta\delta$ is required to compare with the modulus of complex values.

Supplementary References

[1] Steven L. Brunton, Joshua L. Proctor, and J. Nathan Kutz. Discovering governing equations from data by sparse identification of nonlinear dynamical systems. *Proceedings of the National Academy of Sciences*, 113(15):3932–3937, 2016.

[2] Samuel H. Rudy, Steven L. Brunton, Joshua L. Proctor, and J. Nathan Kutz. Data-driven discovery of partial differential equations. *Science Advances*, 3(4):e1602614, 2017.

[3] M. D. McKay, R. J. Beckman, and W. J. Conover. A comparison of three methods for selecting values of input variables in the analysis of output from a computer code. *Technometrics*, 21(2):239–245, 1979.

[4] Xavier Glorot and Yoshua Bengio. Understanding the difficulty of training deep feedforward neural networks. In *Proceedings of the thirteenth international conference on artificial intelligence and statistics*, pages 249–256. JMLR Workshop and Conference Proceedings, 2010.

[5] Hayden Schaeffer. Learning partial differential equations via data discovery and sparse optimization. *Proceedings of the Royal Society A: Mathematical, Physical and Engineering Sciences*, 473(2197):20160446, 2017.

[6] Kunihiko Taira and Tim Colonius. The immersed boundary method: A projection approach. *Journal of Computational Physics*, 225(2):2118–2137, 2007.

[7] Ankur Gupta and Saikat Chakraborty. Linear stability analysis of high- and low-dimensional models for describing mixing-limited pattern formation in homogeneous autocatalytic reactors. *Chemical Engineering Journal*, 145(3):399–411, 2009.

[8] Lionel G Harrison. Kinetic theory of living pattern. *Endeavour*, 18(4):130–136, 1994.

[9] Elizabeth E Holmes, Mark A Lewis, JE Banks, and RR Veit. Partial differential equations in ecology: spatial interactions and population dynamics. *Ecology*, 75(1):17–29, 1994.

[10] Richard FitzHugh. Impulses and physiological states in theoretical models of nerve membrane. *Biophysical journal*, 1(6):445, 1961.

[11] Jinichi Nagumo, Suguru Arimoto, and Shuji Yoshizawa. An active pulse transmission line simulating nerve axon. *Proceedings of the IRE*, 50(10):2061–2070, 1962.

[12] Wang Jin, Esha T Shah, Catherine J Penington, Scott W McCue, Lisa K Chopin, and Matthew J Simpson. Reproducibility of scratch assays is affected by the initial degree of confluence: experiments, modelling and model selection. *Journal of theoretical biology*, 390:136–145, 2016.

[13] Ronald Aylmer Fisher. The wave of advance of advantageous genes. *Annals of Eugenics*, 7(4):355–369, 1937.

[14] Philip K Maini, DL Sean McElwain, and David I Leavesley. Traveling wave model to interpret a wound-healing cell migration assay for human peritoneal mesothelial cells. *Tissue Engineering*, 10(3-4):475–482, 2004.

[15] Krithika Manohar, J. Nathan Kutz, and Steven L. Brunton. Optimal Sensor and Actuator Selection using Balanced Model Reduction. *arXiv e-prints*, page arXiv:1812.01574, December 2018.

[16] I.M Sobol. On the distribution of points in a cube and the approximate evaluation of integrals. *USSR Computational Mathematics and Mathematical Physics*, 7(4):86–112, 1967.

[17] Dongkun Zhang, Lu Lu, Ling Guo, and George Em Karniadakis. Quantifying total uncertainty in physics-informed neural networks for solving forward and inverse stochastic problems. *Journal of Computational Physics*, 397:108850, 2019.

[18] Maziar Raissi, Paris Perdikaris, and George E Karniadakis. Physics-informed neural networks: A deep learning framework for solving forward and inverse problems involving nonlinear partial differential equations. *Journal of Computational Physics*, 378:686–707, 2019.

[19] Zhiping Mao, Ameya D Jagtap, and George Em Karniadakis. Physics-informed neural networks for high-speed flows. *Computer Methods in Applied Mechanics and Engineering*, 360:112789, 2020.

[20] Guofei Pang, Lu Lu, and George Em Karniadakis. fpinns: Fractional physics-informed neural networks. *SIAM Journal on Scientific Computing*, 41(4):A2603–A2626, 2019.

[21] Sifan Wang, Yujun Teng, and Paris Perdikaris. Understanding and mitigating gradient pathologies in physics-informed neural networks. *arXiv preprint arXiv:2001.04536*, 2020.

[22] Francisco Sahli Costabal, Yibo Yang, Paris Perdikaris, Daniel E Hurtado, and Ellen Kuhl. Physics-informed neural networks for cardiac activation mapping. *Frontiers in Physics*, 8:42, 2020.

[23] Yasumasa Nishiura and Daishin Ueyama. Spatio-temporal chaos for the gray–scott model. *Physica D: Nonlinear Phenomena*, 150(3-4):137–162, 2001.
